# Adipose Tissue Gene Expression of Entire Male, Immunocastrated and Surgically Castrated Pigs

**DOI:** 10.3390/ijms22041768

**Published:** 2021-02-10

**Authors:** Klavdija Poklukar, Marjeta Čandek-Potokar, Milka Vrecl, Nina Batorek-Lukač, Gregor Fazarinc, Kevin Kress, Volker Stefanski, Martin Škrlep

**Affiliations:** 1 Agricultural Institute of Slovenia, Hacquetova ulica 17, 1000 Ljubljana, Slovenia; klavdija.poklukar@kis.si (K.P.); meta.candek-potokar@kis.si (M.Č.-P.); nina.batorek@kis.si (N.B.-L.); 2 Faculty of Agriculture and Life Sciences, University of Maribor, Pivola 10, 2311 Hoče, Slovenia; 3 Institute of Preclinical Sciences, Veterinary Faculty, University of Ljubljana, Gerbičeva ulica 60, 1000 Ljubljana, Slovenia; milka.vrecl@vf.uni-lj.si (M.V.); gregor.fazarinc@vf.uni-lj.si (G.F.); 4 Behavioural Physiology of Livestock, Institute of Animal Science, University of Hohenheim, Garbenstrasse 17, 70599 Stuttgart, Germany; kress.kevin@uni-hohenheim.de (K.K.); volker.stefanski@uni-hohenheim.de (V.S.)

**Keywords:** pigs, adipose tissue, entire males, immunocastration, surgical castration, RNA-sequencing, expression

## Abstract

Differences in adipose tissue deposition and properties between pig male sex categories, i.e., entire males (EM), immunocastrates (IC) and surgical castrates (SC) are relatively well-characterized, whereas the underlying molecular mechanisms are still not fully understood. To gain knowledge about the genetic regulation of the differences in adipose tissue deposition, two different approaches were used: RNA-sequencing and candidate gene expression by quantitative PCR. A total of 83 differentially expressed genes were identified between EM and IC, 15 between IC and SC and 48 between EM and SC by RNA-sequencing of the subcutaneous adipose tissue. Comparing EM with IC or SC, upregulated genes related to extracellular matrix dynamics and adipogenesis, and downregulated genes involved in the control of lipid and carbohydrate metabolism were detected. Differential gene expression generally indicated high similarity between IC and SC as opposed to EM, except for several heat shock protein genes that were upregulated in EM and IC compared with SC. The candidate gene expression approach showed that genes involved in lipogenesis were downregulated in EM compared with IC pigs, further confirming RNA-sequencing results.

## 1. Introduction

Castration of male piglets has been practised for centuries, mainly to prevent consumers’ negative response to boar taint. The most common practice is surgical castration performed within the first week of piglets’ life [1] and, therefore, represents an early-life deprivation of male hormones. Lately, surgical castration as practised has been criticised and its further use questioned due to the pain inflicted to the animals during the procedure [2]. The two most viable alternatives are rearing of entire males (EM) or immunocastrated males (IC) [3,4,5]. Entire males are characterised by a high androgenic potential compared to surgically castrated pigs (SC), show a higher capacity for protein and a lower capacity for lipid deposition [6,7], and are, therefore, also more cost-effective. On the other hand, rearing EM is problematic due to more aggressive behaviour, excessive carcass leanness, inferior fat and meat quality of EM, and especially the development of boar taint [8,9]. The second alternative is immunocastration, which is a procedure of immunogenic blocking of testicular function consisting of the vaccination against endogenous gonadotropin-releasing hormone (GnRH) causing a castration-like effect [10,11]. For an effective response, the vaccine should be administered twice. According to the most common vaccination practice, the first dose is administered early in life at around 10–12 weeks of age, and the second dose is administrated at around 19–21 weeks (i.e., 4–6 weeks before slaughter), which denotes that the deprivation of male hormones is effective later than in the case of SC. Before the second vaccination (V2), IC pigs are metabolically equal to EM [11,12], but after V2, their metabolism progressively turns towards more castrate-like, with a notable increase in feed intake and fat tissue deposition [13,14], which increases almost linearly with the time elapsed from the V2 to slaughter [15]. We recently demonstrated [16] that the increase in quantity of fat depots after immunocastration is associated with a larger adipocyte and lobulus surface area in the backfat, together with notably increased activity of lipogenic enzymes (i.e., fatty acid synthase, glucose 6-phosphate dehydrogenase, malic enzyme and citrate cleavage enzyme) and increased fat saturation as a result of the elevated de novo synthesis of palmitic and stearic acids.

Studies addressing the causal molecular processes responsible for the differences in fat deposition between male sex categories are scarce. Moreover, the existing investigations [17,18,19] were merely focused on the comparison between EM and SC pigs. In these studies, the underlying molecular regulations between EM and SC were identified by assessing the differences in gene expression using quantitative PCR (qPCR) [17] or microarray approach [18,19]. Quantitative PCR is highly sensitive and reproducible; however, it can capture the expression of a relatively small number of transcripts, while microarray technology is capable of capturing the expression of the larger, but predefined set of transcripts. Another approach, a high-throughput sequencing technology (RNA-seq), has recently become the preferred method for determination of RNA presence and quantity due to the detection of the large dynamic range of expression levels [20]. In the present study, RNA-seq analysis was performed to find possible molecular mechanisms responsible for the differences in fat deposition between male sex categories. To remove animal-specific differences and to highlight general genes and mechanisms with the highest variance, RNA-seq on pooled RNA samples was performed [21]. However, it is possible that some genes, with lower expression variance, might not be detected when samples are pooled. Therefore, pooled RNA-seq was complemented with a candidate gene expression approach using qPCR on individual animals to discover the expression of the target genes with the known importance for the main lipid metabolic mechanisms. 

The objective of our study was to characterize the underlying molecular processes occurring in the adipose tissue of EM, IC and SC pigs using two approaches: the detection of differentially expressed genes and identification of gene networks using RNA-seq on pooled RNA; validation of differentially expressed genes discovered by RNA-seq as well as measuring the expression of pre-selected candidate genes involved in lipid metabolism on individual animals by qPCR.

## 2. Results

### 2.1. RNA-Sequencing Approach

#### 2.1.1. Description of RNA-seq Data

RNA-seq approach was performed to identify genes and gene pathways responsible for the differences in backfat tissue properties according to male sex category (i.e., EM, IC and SC). The sequencing yielded approximately 89.5 million paired-end raw reads per pool. A total of 77.1, 96.7 and 91.0 million high-quality reads were obtained for EM, IC and SC pig samples, respectively (Supplementary Table S1). Approximately 77% of the clean reads were mapped to the annotated *Sscrofa* 10.2 genome (Ensembl release 89). Of all the mapped clean reads, 72.2–73.03% of reads had unique matches and 4.24–4.53% showed multiple-position matches (Supplementary Table S2). 

#### 2.1.2. Gene Expression Level Analysis

Gene expression level was estimated for each sample. A total of 12,120 genes were expressed in adipose tissue of EM, IC and SC pigs. Among them, 112, 220 and 229 genes were uniquely expressed in EM, IC and SC, respectively (Supplementary Figure S1).

#### 2.1.3. Differential Expression Analysis

**Figure 1:**
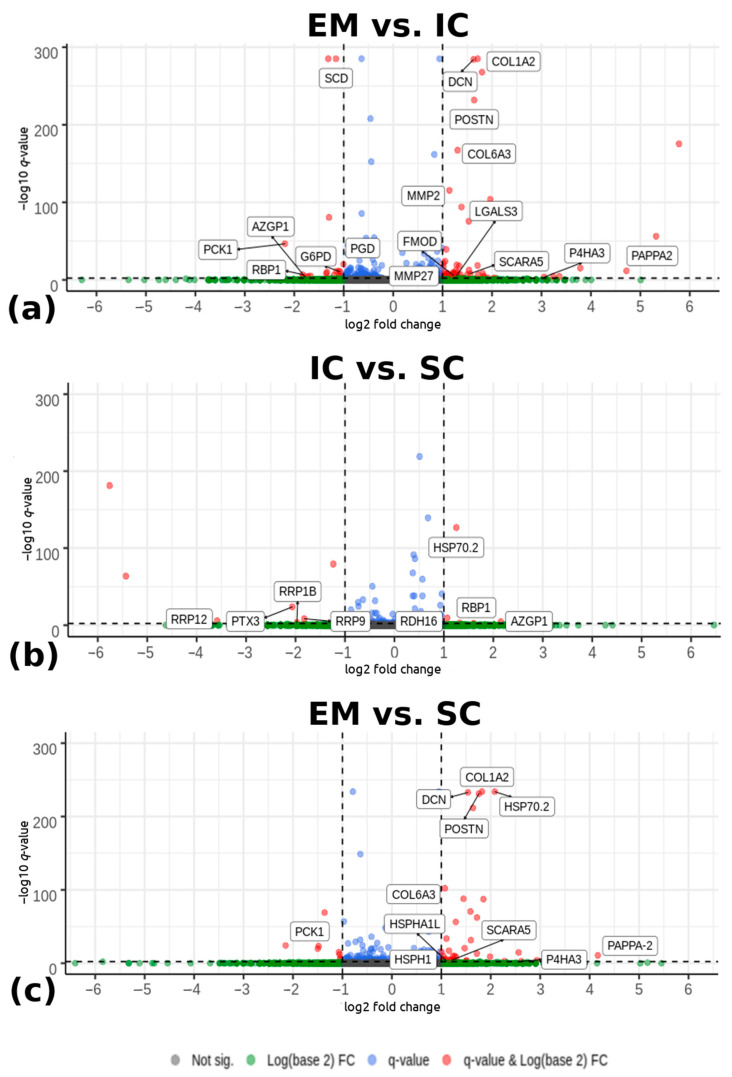
Volcano plots depicting genes expressed in the backfat tissue from pigs according to male sex category: (**a**) EM and IC, (**b**) IC and SC, and (**c**) EM and SC pigs. The horizontal lines indicate the significance threshold of differentially expressed genes at -log_10_(*q*-value) < 0.005. The vertical lines represent the threshold of |log_2_ (fold change) > 1|. Red dots represent upregulated and downregulated differentially expressed genes with |log_2_ (fold change) > 1| and -log_10_(*q*-value) < 0.005. Genes (dots) that are labelled are the most important genes which were identified and discussed in the Discussion chapter. Blue and green dots are representing the remaining detected genes that did not meet the determined criteria. EM = entire males; IC = immunocastrated pigs; SC = surgically castrated pigs; FC = fold change.

Differential expression analysis detected 83 differentially expressed genes between EM and IC with |log_2_(fold change) > 1| and −log_10_(*q*-value) < 0.005 (Figure 1a). Among them, 60 genes were upregulated and 23 were downregulated in EM compared with IC. Comparing IC and SC (Figure 1b), 15 significantly differentially expressed genes were detected. Among them, six genes were upregulated and nine were downregulated in IC compared with the SC. There were 48 differentially expressed genes between EM and SC, with 40 genes upregulated and 8 genes downregulated in EM compared with SC. The Volcano plot (Figure 1) shows differentially expressed genes between EM and IC, IC and SC, and EM and SC. The complete lists of all identified differentially expressed genes with their respective fold changes *p*- and *q*-values are given in Supplementary Table S3.

**Figure 2:**
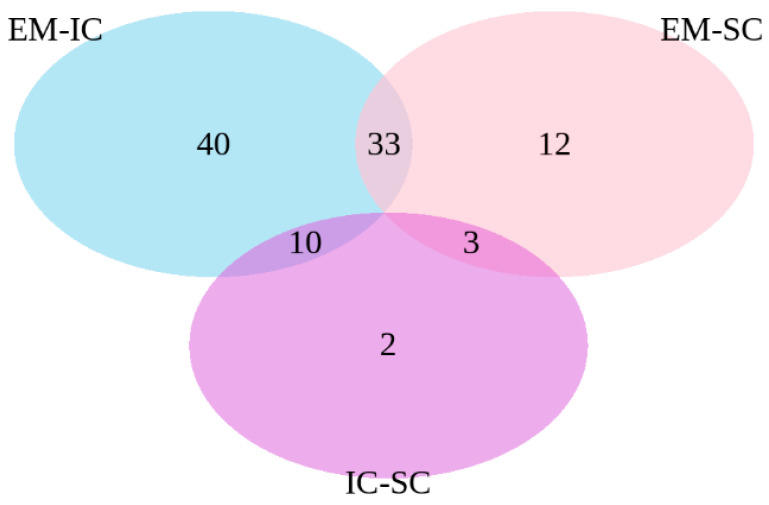
Venn diagram of differentially expressed genes between EM vs. IC, IC vs. SC and EM vs. SC. EM = entire males; IC = immunocastrated pigs; SC = surgically castrated pigs.

The common differentially expressed genes were identified in the pair-wise comparisons between sex categories (Figure 2). The comparisons of EM vs. IC and EM vs. SC showed 33 common genes, the comparisons EM vs. IC and IC vs. SC showed 10 common genes, while the comparisons EM vs. SC and IC vs. SC showed three common genes.

**Figure 3:**
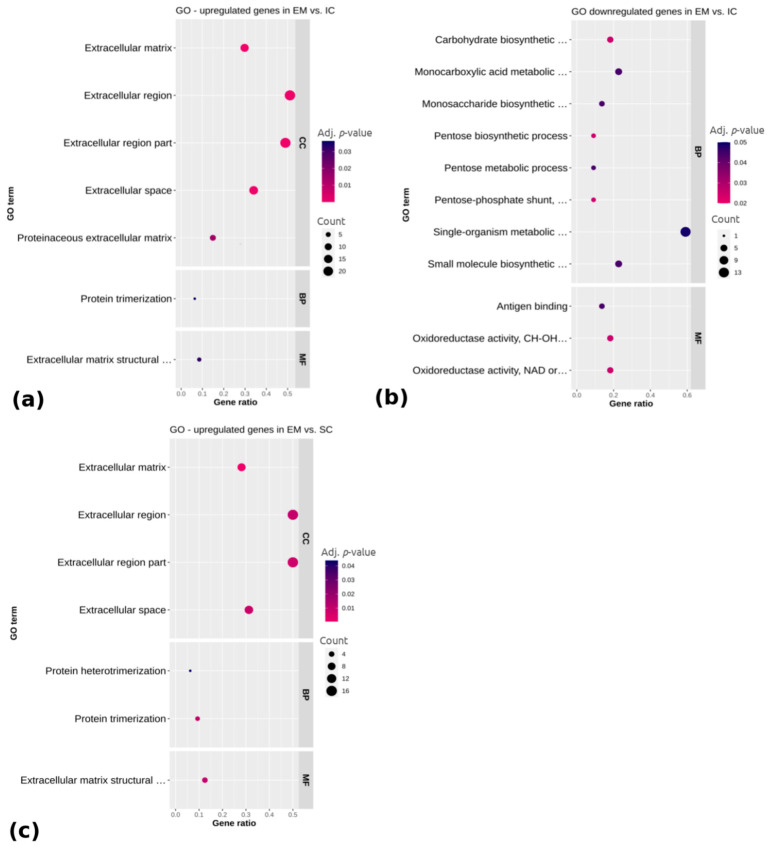
Functional categorization of differentially (**a**) upregulated and (**b**) downregulated genes between EM and IC and (**c**) upregulated genes between EM and SC. Gene ontology (GO) enrichment analysis is representing cellular component category (CC), biological process category (BP) and molecular function category (MF). Gene ratio is the ratio between differentially expressed genes and total differentially expressed genes in the given GO term. EM = entire males; IC = immunocastrated pigs; SC = surgically castrated pigs.

**Figure 4:**
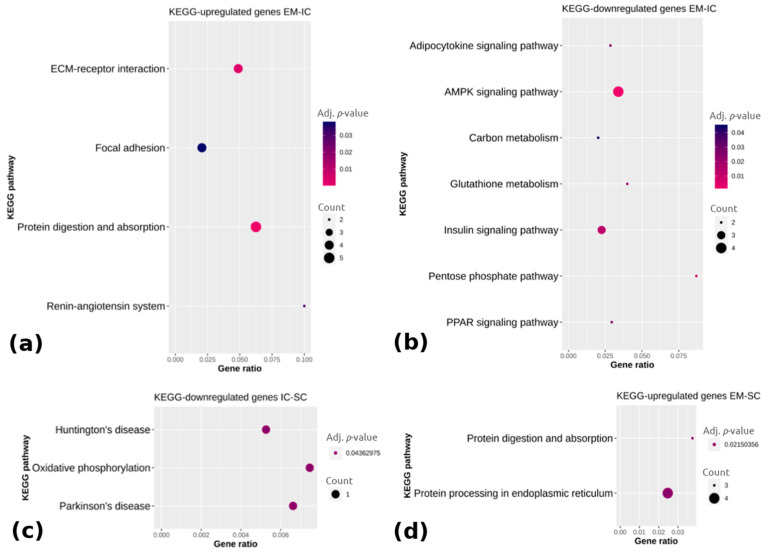
KEGG (Kyoto Encyclopedia of Genes and Genomes) pathway enrichment analysis of (**a**) upregulated and (**b**) downregulated genes between EM and IC, (**c**) downregulated genes between IC and SC and (**d**) upregulated genes between EM and SC. Gene ratio is the ratio between differentially expressed genes and total differentially expressed genes in the given KEGG pathway. EM = entire males; IC = immunocastrated pigs; SC = surgically castrated pigs; ECM = extracellular matrix; AMPK = adenosine monophosphate-activated protein kinase; PPAR = peroxisome proliferator activated receptor.

To annotate differentially expressed genes related to the adipose tissue regulation in different male sex categories, the GO (Gene ontology) (Figure 3) and KEGG (Kyoto Encyclopedia of Genes and Genomes) pathways (Figure 4) were analyzed for the upregulated and downregulated genes, respectively. Regarding the genes upregulated in EM compared with IC (Figure 3a), the GO terms were mainly related to the extracellular region, including the extracellular matrix, extracellular space, extracellular region part and proteinaceous extracellular matrix (adj. *p* < 0.05), while the downregulated genes were related to monocarboxylic acid metabolic process, monosaccharide biosynthetic process, single organism metabolic process, small molecule metabolic process and molecular function of oxidoreductase activity (adj. *p* < 0.05). No significantly enriched GO terms or pathways were found for the genes that were differentially expressed between IC and SC. Concerning the upregulated genes in EM compared with SC pigs, there were seven significantly enriched GO terms (Figure 3c). A total of four of them belonged to the cellular component category and were, as in the case of EM vs. IC comparison, mostly associated with the extracellular region. Molecular function category also contained the term extracellular matrix structural constituent, while the biological process category contained the terms protein trimerization and protein heterotrimerization (adj. *p* < 0.05). No significantly enriched terms were found for the downregulated genes in EM compared with SC pigs. The complete lists of top 15 identified GO terms with corresponding differentially expressed genes are reported in Supplementary Table S4.

Pathway analysis showed that the upregulated genes in EM compared with IC (Figure 4a) were associated with the extracellular matrix receptor interaction, focal adhesion, protein digestion and absorption and the renin-angiotensin system (adj. *p* < 0.05), whereas the downregulated genes in EM vs. IC (Figure 4b) were mainly enriched in the adipocytokine signaling pathway, adenosine monophosphate-activated protein kinase (AMPK) signaling pathway, carbon metabolism, insulin signaling pathway and peroxisome proliferator-activated receptor signaling pathway (adj. *p* < 0.05). The downregulated genes in IC vs. SC were in three pathways, with the most relevant one being oxidative phosphorylation (adj. *p* < 0.05). Comparing EM with SC, two pathways were enriched (i.e., protein digestion and absorption and protein processing in the endoplasmic reticulum) based on the upregulated genes in EM (adj. *p* < 0.05), whereas no pathways could be identified in the case of the downregulated genes in EM. The complete lists of the top 15 enriched pathways with differentially expressed genes are reported in Supplementary Table S5.

### 2.2. Gene Expression Using Quantitative PCR Approach

**Figure 5:**
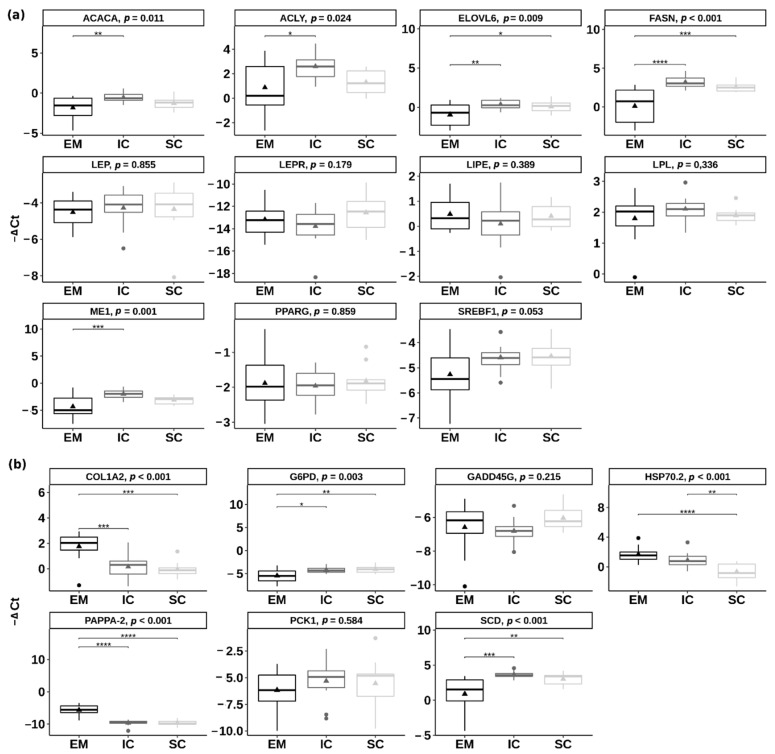
Differential expression of (**a**) the candidate genes involved in lipid metabolism and (**b**) the genes detected in the RNA-sequencing approach between entire males (EM), immunocastrated pigs (IC) and surgically castrated pigs (SC) in the inner backfat layer. The results are presenting ΔCt value of target transcripts derived using the comparative Ct method (ΔCt = Ct _geometric mean of controls_ − Ct _target transcript_). Larger ΔCt represents higher gene expression. Triangles are representing the mean ΔCt value. In the case of statistical significance (reported as Tukey adjusted *p*-value), the asterisks are drawn (**** *p* < 0.0001, *** *p* < 0.001; ** *p* < 0.01; * *p* < 0.05). ACACA = acetyl CoA carboxylase; ACLY = ATP citrate lyase; COL1A2 = collagen type I alpha 2 chain; ELOVL6 = ELOVL fatty acid elongase 6; FASN = fatty acid synthetase; G6PD = glucose-6-phosphate dehydrogenase; GADD45G = growth arrest and DNA damage inducible gamma; HSP70.2 = heat shock protein family A member 1B; LEP = leptin; LEPR = leptin receptor; LIPE = lipase E; LPL = lipoprotein lipase; ME1 = malic enzyme 1; PAPPA-2 = pappalysin 2; PCK1 = phosphoenolpyruvate carboxykinase 1; PPARγ = peroxisome proliferator activated receptor gamma; SCD = stearoyl-CoA desaturase; SREBF1 = sterol regulatory element binding transcription factor 1.

Relative expression of the eleven pre-selected candidate genes involved in lipid metabolism and energy homeostasis was quantified in the backfat of EM, IC and SC. The genes ACACA (acetyl-CoA carboxylase), ACLY (ATP citrate lyase), FASN (fatty acid synthase), ELOVL6 (ELOVL fatty acid elongase 6) and ME1 (malic enzyme 1) had significantly lower expression (*p* < 0.05) (Figure 5a) in EM than IC, which ranged from 2.44 (log_2_ fold change = 1.28) for ACACA gene to 8.48 (log_2_ fold change = 3.08) for FASN gene. There were no significant differences between IC and SC in the expression of the pre-selected candidate genes (*p* > 0.05). The genes FASN and ELOVL6 were downregulated in EM compared with SC (*p* < 0.05).

Among the genes that were chosen based on the RNA-seq results (i.e., COL1A2—collagen type I alpha 2 chain, G6PD—glucose-6-phosphate dehydrogenase, GADD45G—growth arrest and DNA damage inducible gamma, PAPPA-2—pappalysin 2, PCK1—phosphoenolpyruvate carboxykinase 1, SCD—stearoyl-CoA desaturase and HSP70.2—heat shock protein family A member 1B), several differed significantly also using the qPCR approach (Figure 5b). The genes COL1A2 and PAPPA-2 were upregulated in EM compared with IC and SC (*p* < 0.001), the gene HSP70.2 was upregulated in EM and IC compared with SC, while the genes G6PD and SCD were downregulated in EM compared with IC and SC (*p* < 0.01). Most of the genes were regulated in the same direction in RNA-seq and qPCR approaches, except the GADD45G gene for which no significant differences were found between any of the pair-wise comparisons in the case of qPCR validation (Supplementary Figure S2).

## 3. Discussion

### 3.1. RNA-Sequencing Approach

In the present study, several gene networks and underlying genes responsible for the differences in backfat deposition between the three male sex categories (i.e., EM, IC and SC) were identified. Functional enrichment analysis showed that the upregulated genes in EM as compared with IC and SC were significantly enriched in extracellular region/matrix cellular components, extracellular matrix receptor interaction and focal adhesion pathways. For instance, this includes genes involved in the synthesis of collagen (e.g., COL1A2, COL6A3), which are the major components of the extracellular matrix (ECM) [22]. It has been demonstrated in different tissues that EM, due to the effect of androgens, have more collagen than castrates [23]; in that respect, IC were shown to be in-between EM and SC [16,24]. Similarly, in cattle, downregulation of genes related to collagen synthesis (including COL1A2) in subcutaneous adipose tissue and muscle have been related to castration [25,26]. Furthermore, in a present study, a key enzyme for collagen synthesis, i.e., P4HA3 (prolyl 4-hydroxylase subunit alpha), was upregulated in EM as compared with IC and SC pigs. This gene encodes a component of prolyl 4-hydroxylase catalyzing the formation of 4-hydroxyproline, which is essential for folding of newly synthesized procollagen chains [27]. In our study, several other genes, coding matrix proteins regulating collagen fibrillogenesis, were also upregulated in the EM compared with IC or SC. This accounts for genes encoding proteoglycans, i.e., DCN (decorin) and FMOD (fibromodulin), which are required for proper collagen folding and ECM stabilization through the interaction with several molecules present in the ECM [28,29,30], and POSTN (periostin), which is a mediator of the biomechanical properties of the connective tissue [31]. The expression of the POSTN gene is crucial for the collagen cross-linking and maintenance of the ECM [32,33]. Some other genes involved in the ECM degradation (i.e., matrix metalloproteinases MMP2 and MMP27) were in the present study also upregulated in EM as compared with IC, although the level of expression difference was relatively low. Thus, it can be hypothesized that EM have a more developed or denser connective tissue within fat depots due to a higher synthesis and remodulation of the ECM, which is supported by the upregulation of the genes related to ECM in EM as compared with IC and SC.

Large differences in expression, between EM and both groups of castrated pigs, were found for the PAPPA-2 gene, which was downregulated in IC and SC. The pappalysin 2 enzyme cleaves insulin-like growth factor 1 (IGF-1) in ternary complex with insulin-like growth factor binding proteins (IGFBPs), thereby releasing IGF-1 and consequently leading to the increased IGF-1 bioavailability [34,35,36]. Insulin-like growth factor 1 has been implicated in the proliferation and differentiation of adipocytes [37]. Higher levels of serum IGF-1 were also reported for EM than SC [11,38], whereas in IC pigs, the levels of IGF-1 start to decrease after the effective immunization. Stable IGF-1 level is in IC pigs (compared to that of SC) reached within 5 to 10 days after V2 [10]. Therefore, downregulation of PAPPA-2 expression in IC and SC pigs is indicative of increased binding of IGFBPs with IGF-1 and consequently inhibition of IGF-1 action in castrated animals, which affects adipocyte proliferation and differentiation. 

Adipose tissue of EM contains smaller adipocytes than adipose tissue of IC and SC, while there seem to be no differences in the number of adipocytes between EM, IC and SC [16]. However, the present research identified a higher expression of the genes involved in adipocyte proliferation and differentiation (i.e., SCARA5—scavenger receptor class A member 5, LGALS3—galectin 3, RBP1—retinol binding protein 1) in EM than IC or SC. For instance, SCARA5 gene, which mediates the early stage of mesenchymal cell lineage commitment into adipocytes [39], was upregulated in EM compared with IC and SC. Similarly, LGALS3 gene, which was also upregulated in EM compared with IC, has been found to activate the expression of the major transcription factor for adipocyte differentiation, i.e., peroxisome proliferator activated receptor gamma—PPARγ [40]. In accordance, retinol-binding protein 1, which is the inhibitor of the PPARγ gene [41], was downregulated in EM compared with IC and SC. Although the RNA-seq results for EM indicated overexpression of some genes involved in adipogenesis (i.e., SCARA5, LGALS3, RBP1), the expression of the PPARγ gene, recognized as a master regulator of adipogenesis and most abundantly expressed in adipose tissue [42], did not differ between the sex categories. 

With the smaller adipocytes observed in EM than IC and SC pigs [16], we observed a downregulation of the PCK1 and SCD genes (both involved in monocarboxylic metabolic process, small molecule biosynthetic process, PPAR signaling and AMPK signaling pathways) in EM compared with both IC and SC. Phosphoenolpyruvate carboxykinase 1 enzyme is involved in glyceroneogenesis and reesterification of free fatty acids in white adipose tissue [43], and the changes in PCK1 expression and activity were previously associated with the fat quantity [44,45,46,47,48]. Similarly, SCD expression has also been demonstrated to regulate lipid deposition and metabolism. The expression of the SCD gene is induced in differentiated adipocytes [49]. The SCD gene is responsible for the biosynthesis of monounsaturated fatty acids (MUFA) from the saturated fatty acids (SFA). Fatty acid composition data of the backfat demonstrated that IC and SC have increased MUFA content compared to EM [16,24]. Since SCD expression is under the hormonal (e.g., leptin, insulin, androgens) or nutritional regulation [50], the respective differences between male sex categories [11,12] corroborate with the upregulated expression of SCD. Functional enrichment analysis showed a downregulation of some genes (i.e., G6PD, PGD, GYS2, PCK1) in EM vs. IC pointing on differences between them in biological processes related to carbohydrate metabolism (e.g., monosaccharide biosynthetic process, carbohydrate biosynthetic process). This result is consistent with the findings of the authors Floc’h et al. [51], who observed that immunocastration affects energy metabolism much faster than protein metabolism and that glucose clearance after meal intake in IC pigs is faster than in EM. This is also consistent with the evidence that carbohydrates are used for lipogenesis with higher intensity in IC after V2, while the protein deposition remains similar [12]. Another interesting differentially expressed candidate gene involved in lipid metabolism was the adipokine zinc-alpha-2-glycoprotein (AZGP1). This gene was downregulated in EM and SC compared with IC. The AZGP1 gene stimulates lipid degradation in adipocytes and is, therefore, considered a lipid-mobilizing factor [52]. It was suggested that AZGP1 is part of a mechanism by which growth hormone (GH) modulates subcutaneous adipose tissue lipid metabolism indicating a potential role for GH-AZGP1 axis in protecting against the development of obesity [53]. The upregulated expression of this gene in IC may be indicative of a specific IC metabolism after V2, but this could also depend on vaccination timing.

Several members of the heat shock protein family 70 (HSP70, i.e., HSP70.2, HSPA1L, HSPH1) were overexpressed in EM and one of them also in IC (i.e., HSP70.2) compared with SC pigs. Heat shock proteins act as protein chaperons during the protein assembly, protein folding and unfolding, refolding or degradation of damaged proteins, protein translocation and maintenance of structural proteins. A variety of stress factors (for instance exercise) can affect the overexpression of heat shock proteins [54], which is in line with a higher level of activity and aggressive behavior reported for EM than SC [55,56]. A higher protein abundance of HSP70 proteins in EM than SC pigs was also reported in a recent proteomic study [57]. Immunocastrates (after the effective immunization) reduce their activity and aggressive behavior to the level similar to SC [58], although some factors like feed restriction could still increase aggressiveness and stress response in IC pigs [14]. Overexpression of the HSP70.2 in EM and IC in the present study could be also related to the quantity of fat depots as indicated by [59]. Additionally, a polymorphism in the HSP70.2 gene was also reported to be associated with backfat thickness in pigs [60]. 

Regarding the overall similarities between the male sex categories, the number of differentially expressed genes was the lowest when IC was compared with SC. Moreover, 33 genes were common in comparison of EM vs. IC and EM vs. SC. This suggests that castration, regardless of the method used, similarly affects gene expression. This is also consistent with our previous study on the same pigs, where the histo-morphological characteristics of adipose tissue were 5 weeks after V2 similar in IC to SC pigs [16]. 

### 3.2. Candidate Gene Expression Approach

In general, we observed lower expression of genes FASN, ME1, G6PD, ACLY, ACACA involved in the lipogenesis in EM than IC or lower expression of genes FASN and ELOVL6 in EM than SC, which agrees with the increased lipid deposition in castrated pigs. For instance, the FASN gene, which was downregulated in EM compared with IC and SC, is responsible for catalyzing the conversion of malonyl-CoA into palmitate [61]. Several other studies showed that the presence of androgens downregulates the FASN gene expression [17,19] or FASN protein abundance [62], which corroborates with the commonly known effect of androgens on body fat deposition [63]. The lower capacity for fatty acid synthesis in EM than IC was also confirmed by other genes involved in the fatty acid synthesis, including the ones providing the energy for reductive biosynthesis (ME1 and G6PD), supplying acetyl-CoA for fatty acid biosynthesis (ACLY) and catalyzing carboxylation of acetyl-CoA to malonyl-CoA (ACACA) [61]. Our previous study on the same animals showed that the activities of fatty acid synthase, malic enzyme, glucose-6-phosphate dehydrogenase and ATP citrate lyase enzymes were significantly lower in the backfat of EM than IC, indicating a rapid increase in fat deposition only 5 weeks after V2, whereas in SC, other factors (e.g., inhibitors) may cause lower lipogenic activity [16]. Another gene that was downregulated in EM compared with IC and SC was ELOVL6, which is responsible for the elongation of SFA and MUFA with 12, 14, and 16 carbon atoms. Loss of ELOVL6 function increases the level of palmitic acid and reduces the level of stearic and oleic acid [64]. In agreement with this, data on the fatty acid composition of backfat showed that the content of stearic and oleic acid was the lowest in EM and the highest in SC pigs, with the inconsistent placement of IC [16,24]. In the present study, differential gene expressions of FASN, ME, ACACA, ACLY and ELOVL6 were not initially detected with the RNA-seq approach. The detection of additional differentially expressed genes using qPCR may be indicative of a higher sensitivity of qPCR than RNA-seq [65] and/or using pooling technology for RNA-seq. 

## 4. Material and Methods

### 4.1. Animals and Sample Collection

The animals used in this study were obtained from a trial conducted within ERA-NET SusAn project SuSI (grant number 696231). The trial was approved by the Ethical committee of the Regional council of Tübingen, Germany (project identification number HOH 47/17^TH^, 22/08/2017) described in detail by [66]. Briefly, 36 pigs originating from one slaughter batch (12 EM, 12 IC and 12 SC) of the commercial Landrace x Pietrain crosses were used. Surgically castrated pigs were castrated within the first week of life, while IC were vaccinated at the age of 12 and 21 weeks with a vaccine against GnRH (Improvac**^®^**vaccine, Zoetis Germany, Berlin, Germany). All animals had ad libitum access to the same commercial diet (described in details by [24]). At the age of 26 weeks and the bodyweight of 121.7 ± 1.6 kg (mean ± SEM), animals were slaughtered according to the standard abattoir procedure. Within the first thirty minutes after slaughter, carcasses were split apart, the inner layer of subcutaneous fat at withers (i.e., between first thoracal and last cervical vertebra) was sampled, immediately frozen in liquid nitrogen and stored at −80 °C for subsequent RNA extraction.

### 4.2. RNA Extraction and Quality Check

Total RNA was extracted from approximately 90 mg of frozen backfat tissue using RNeasy plus Universal Mini Extraction Kit (Qiagen, Crawley, UK) according to the manufacturer’s instructions. The concentration and quality of the isolated RNA were validated by measuring the ratio of optical densities (OD) at 260 and 280 nm using NanoPhotometer spectrophotometer (IMPLEN, Westlake Village, CA, USA). The integrity of the RNA (RIN) samples was further assessed using 1% agarose electrophoresis and Agilent 2100 Bioanalyzer (Agilent, Santa Clara, CA, USA). Samples with the OD260/OD280 ratio between 1.7 and 2.1 and RIN value more than 6.3 were used for RNA-seq and qPCR analysis.

### 4.3. cDNA Library Construction and RNA-Sequencing

Isolated RNA samples were pooled with equal quantities in each sex category (i.e., EM, IC and SC groups) and cDNA sequencing libraries were generated using NEBNext Ultra RNA Library preparation Kit for Illumina (NEB, Ipswich, MA, USA) according to the manufacturer’s instructions. Briefly, mRNA was extracted using poly-T oligo (dT) magnetic beads. The enriched mRNAs were fragmented using divalent cations under elevated temperature in NEBNext First Strand Reaction Buffer (5×), followed by synthesis of the first cDNA strand using random hexamer primer and M-MuLV Reverse Transcriptase with reduced RNase H activity, and the second strand cDNA synthesis using second-strand synthesis reaction buffer with enzyme mix (i.e., DNA polymerase I, RNase H). Remaining overhangs were blunted using exonuclease/polymerase. Afterwards, adenylation of 3′ ends of the cDNA fragments and ligation of NEBNext adaptors was performed, followed by the purification step using AMPure XP System (Beckman Coulter, Beverly, MA, USA) and size selection step using USER enzyme (NEB, Ipswich, MA, USA). Enrichment of the cDNA libraries was performed with PCR. Obtained PCR products were further purified using AMPure XP System (Beckman Coulter Life Sciences, Indianapolis, IN, USA) and quantified using the Agilent Bioanalyzer 2100 System (Agilent, Santa Clara, CA, USA). Libraries of the different index-coded samples were clustered on a cBot Cluster Generation System (Illumina, San Diego, CA, USA) and sequenced on an Illumina Hiseq (Illumina, San Diego, CA, USA) generating 150 bp paired-end reads (Novogene Bioinformatics Technology Co., Ltd., Beijing, China). The RNA-seq experiment was submitted to the NCBI Gene Expression Omnibus (GEO) (Bethseda, MD, USA) and assigned the GEO accession number GSE164391.

### 4.4. Bioinformatic Analysis

#### 4.4.1. Quality Control, Mapping and Assembly

The raw sequencing data were filtered by Novogene Bioinformatics Technology Co., Ltd. (Beijing, China) through in-house perl scripts by (1) discarding the reads with adaptor contamination, (2) removing the reads with more than 10% of uncertain nucleotides and (3) discarding reads with low-quality nucleotides (base quality less than 20) constituting more than 50% of the read. The error rate (%), quality scores (Q20 and Q30 values) and GC-content (%) of the resulting high-quality clean reads were evaluated. Subsequently, clean reads were aligned against the Ensembl reference genome *Sscrofa* 10.2 (Ensembl release 89) using TopHat2 v. 2.0.12 [67] by calling Bowtie v. 2.2.3 [68] with the default parameters allowing up to 2 bases mismatches. 

#### 4.4.2. Quality Control, Mapping and Assembly

HTSeq v. 0.6.1 software [69] was used to count the reads number mapped to each gene. Afterwards, the expected number of Fragments per Kilobase of transcript sequence per Millions of base pairs sequenced was calculated for each gene. 

For this experiment comparing different male sex categories without replicates, the read counts were adjusted by the trimmed mean of M-values (TMM) using edgeR R package (v. 3.22.0) [70]. Identification of differentially expressed genes comparing two groups (i.e., EM vs. IC, IC vs. SC, EM vs. SC) was performed using the DEGSeq R package (v. 1.20.0), which integrates Fisher’s exact test and likelihood ratio test to perform differential expression analysis following a binomial distribution [71]. The resulting *p*-values were adjusted using the Benjamini-Hochberg method. Significant differential expression threshold was set at the corrected *q*-value of 0.005 and |log_2_ (Fold change)| of 1. Volcano plot was created using EnhancedVolcano R package v. 1.8.0 [72].

#### 4.4.3. Functional Enrichment Analysis

Gene ontology (GO) analysis of differentially expressed genes was performed by the R package GOSeq (v. 1.0.3) [73] with the correction of gene lengths. Gene ontology terms with the corrected *p*-values below 0.05 were considered significantly enriched. Additionally, KOBAS software [74] was used to test the statistical enrichment of differentially expressed genes in the Kyoto Encyclopedia of Genes and Genomes (KEGG) pathways using Fisher’s exact test. Test results were subjected to multiple testing correction of the *p*-values by Benjamini and Hochberg correction.

#### 4.4.4. Candidate Genes Expression Analysis by Quantitative PCR

Total RNA per individual pig was used to validate the RNA-seq results and to measure the expression of the selected candidate genes (i.e., genes related to lipid metabolism). Seven genes were selected from the list of differentially expressed genes detected in RNA-seq data, while the other candidate genes were chosen based on their key role in lipid metabolism or energy homeostasis (Table 1). Primers and fluorescent 6-FAM dye-labelled minor groove binder probes/predesigned assays were obtained from Applied Biosystems. Taq-man probes for the pig PAPPA-2 and G6PD genes were designed with Custom TaqMan Assay Design Tool using predicted mRNA *Sus scrofa* sequence (reference NCBI sequence for PAPPA-2: XM_003130330.6 and G6PD: XM_021080744.1). Candidate genes with corresponding assay IDs or primer sequences are given in Table 1.

The qPCR was performed using the TaqMan Universal PCR Master Mix II (Applied Biosystems, Waltham, MA, USA) in the Applied Biosystems 7500 Fast Real-time PCR System. Reaction parameters were identical for all of the tested genes. Briefly, 1.5 µg of the total RNA from individual animals was used for reverse transcription using High-capacity cDNA Reverse Transcription Kit (Applied Biosystem, CA, USA) following the manufacturer’s instructions. Prior to the qPCR analysis, cDNA samples were diluted 10-fold. As negative controls, mixes without cDNA were used. Quantitative PCR was performed in triplicates with the following cycling conditions: one cycle of 50 °C for 2 min, one cycle of 95 °C for 10 min, and 40 cycles of amplification (15 sec at 95 °C and 1 min at 60 °C). The PCR efficiency of each gene was defined using standard curves composed of the three 10-fold cDNA dilutions. 

Peptidylprolyl isomerase A (PPIA), DNA Topoisomerase II Beta (TOP2B) and Beta-2 microglobulin (B2M) (Table 1) were selected as endogenous controls for data normalization with geometric averaging [75]. Quantification of target transcripts, normalized against the geometric mean of PPIA, TOP2B and B2M, was performed according to the comparative Ct method (ΔCt = Ct _geometric mean of controls−_Ct _target transcript_). Normalized qPCR data were analyzed using one-way ANOVA tests in R software with a sex category as a fixed effect. Relative changes in the expression of the studied target transcripts (log_2_ fold changes in the expression) between EM and IC, EM and SC and IC and SC were determined by the comparative 2^− ΔΔ Ct^ method [76]. 

## 5. Conclusions

In summary, RNA-seq and candidate gene expression approaches revealed differences in expression between EM, IC and SC pigs. Specifically, castration of male pigs, regardless of the method, resulted in downregulation of genes involved in ECM dynamics and genes related to adipogenesis. Accordingly, one of the most differentiated candidate gene detected in EM compared to castrates was the PAPPA-2 gene, which affects IGF-1 bioavailability and thus influences adipocyte proliferation and differentiation. The downregulated genes between EM and castrates were involved in lipid and carbohydrate metabolism, consistent with the reduced expression of genes involved in lipogenesis detected using the candidate gene expression approach. Differential expression of heat shock family 70 genes could be a consequence of the more stressful conditions to which EM and IC pigs are exposed. 

## Figures and Tables

**Table 1:** Taq-man probes/primers with corresponding metabolic functions used in the study.

Metabolic Function	Full Gene Name	Gene Symbol	Primer Sequence ^1^ or Taq-Man Assay ID ^2^	Amplicon Length ^3^	RNA-Seq or Reference ^4^
Lipogenesis	Fatty acid synthase	FASN	Ss03386194_u1	95	[17]
	Glucose-6-phosphate dehydrogenase	G6PD	F: GCTTTCCATCAGTCGGATACACATA R: GAACAGCCACCACAGGGT P: CAAGTCGCCCGATGCT	96	RNA-seq
	Malic enzyme 1	ME1	Ss03374853_m1	92	[16]
	ATP citrate lyase	ACLY	Ss03386194_u1	69	[16]
	Acetyl-CoA carboxylase	ACACA	Ss03389963_m1	61	[17]
	Stearoyl-CoA desaturase	SCD	Ss03392313_m1	65	RNA-seq
	ELOVL fatty acid elongase 6	ELOVL6	Ss06879466_m1	90	[77]
Lipid and carbohydrate metabolism	Phosphoenolpyruvate carboxykinase 1	PCK1	Ss03390599_g1	65	RNA-seq
Adipogenesis	Peroxisome proliferator activated receptor gamma	PPARγ	Ss03394829_m1	72	[77]
	Sterol regulatory element binding transcription factor 1	SREBF1	Ss03382914_u1	102	[77]
Lipolysis	Lipoprotein lipase	LPL	Ss03394612_m1	66	[78]
	Lipase E	LIPE	Ss04955671_mH	65	[79]
Energy homeostasis	Leptin	LEP	Ss03392404_m1	68	[80]
	Leptin receptor	LEPR	Ss03379257_u1	142	[81]
Collagen synthesis	Collagen type I alpha 2 chain	COL1A2	Ss03375009_u1	76	RNA-seq
Response to stress	Growth arrest and DNA damage inducible gamma	GADD45G	Ss04246860_g1	54	RNA-seq
Protein protection from the oxidative stress	Heat shock protein family A member 1B	HSP70.2	Ss03392270_g1	69	RNA-seq
Proteolysis against IGFBPs	Pappalysin 2	PAPPA-2	F: CGGAGGGAGGACAGAACAG R: TCACTGATTGTGTGGGAGCAA P: ACACACCTGCAATGAT	70	RNA-seq
Endogenous control	Beta-2 microglobulin	B2M	Ss03391154_m1	60	[82]
	Peptidylprolyl isomerase A	PPIA	Ss03394782_g1	91	[83]
	DNA Topoisomerase II Beta	TOP2B	Ss04953704_m1	61	[84]

^1^ F = forward primer (5′→3′), R = reverse primer (5′→3′), P = probe; ^2^ https://www.thermofisher.com/si/en/home/life-science/pcr/real-time-pcr/real-time-pcr-assays/taqman-gene-expression/single-tube-taqman-gene-expression-analysis.html; ^3^ Amplicon length is in base pairs; ^4^ Selected based on RNA-sequencing results or according to the literature.

## Data Availability

The data presented in this study are openly available in GEO database under the accession number GSE164391.

## Supplementary Materials

The following are available online at https://www.mdpi.com/1422-0067/22/4/1768/s1.
