# Supplementary material for: Adipose Tissue Gene Expression of Entire Male, Immunocastrated and Surgically Castrated Pigs
Source: Int J Mol Sci. 2021 Feb 10;22(4):1768. doi: 10.3390/ijms22041768 (PMC7916650; doi:10.3390/ijms22041768)
Supplement: Supplementary file 1 [file ijms-22-01768-s001.zip › Supplementary_Table_S5.docx]

**Supplementary Table S5: KEGG enrichment analysis** **of the upregulated and downregulated genes between entire males and immunocastrated pigs, between immunocastrated and surgically castrated pigs and between entire males and surgically castrated pigs.**

**Supplementary Table S5.** Kyoto Encyclopedia of Genes and Genomes (KEGG) pathway enrichment analysis of the upregulated and downregulated genes between entire males and immunocastrated pigs, between immunocastrated and surgically castrated pigs and between entire males and surgically castrated pigs.

| **Term** | **ID** | | | | **P-Value** | **Corrected P-Value** | | | **Gene name** |
| --- | --- | --- | --- | --- | --- | --- | --- | --- | --- |
| **KEGG pathways (top 15) of the upregulated genes in entire males compared to immunocastrated pigs** | | | | | | | | | |
| Protein digestion and absorption | ssc04974 | | | | 6.4054E-06 | 0.000301054 | | | COL12A1, COL14A1, COL1A2, COL6A1, COL6A3 |
| ECM-receptor interaction | ssc04512 | | | | 0.00014319 | 0.003364906 | | | COL1A2, COL6A1, COL6A3, ENSSSCG00000014118 |
| Renin-angiotensin system | ssc04614 | | | | 0.00206278 | 0.032316851 | | | ANPEP, AGTR1 |
| Focal adhesion | ssc04510 | | | | 0.00323737 | 0.038039085 | | | COL1A2, COL6A1, COL6A3, ENSSSCG00000014118 |
| Retinol metabolism | ssc00830 | | | | 0.01285868 | 0.104183928 | | | ENSSSCG00000030522, CYPB22 |
| Arginine and proline metabolism | ssc00330 | | | | 0.01330008 | 0.104183928 | | | P4HA3, P4HA2 |
| Chemical carcinogenesis | ssc05204 | | | | 0.01560427 | 0.104771555 | | | ENSSSCG00000030522, CYPB22 |
| PI3K-Akt signaling pathway | ssc04151 | | | | 0.01909179 | 0.112164293 | | | COL1A2, COL6A1, COL6A3, ENSSSCG00000014118 |
| Complement and coagulation cascades | ssc04610 | | | | 0.02232225 | 0.116571739 | | | CZ, ENSSSCG00000015663 |
| Tyrosine metabolism | ssc00350 | | | | 0.09932514 | 0.348572118 | | | ENSSSCG00000030522 |
| Prion diseases | ssc05020 | | | | 0.10209417 | 0.348572118 | | | C7 |
| Bladder cancer | ssc05219 | | | | 0.1048548 | 0.348572118 | | | MMP2 |
| Proteoglycans in cancer | ssc05205 | | | | 0.11966735 | 0.348572118 | | | DCN, MMP2 |
| Fatty acid degradation | ssc00071 | | | | 0.12124374 | 0.348572118 | | | ENSSSCG00000030522 |
| Drug metabolism - cytochrome P450 | ssc00982 | | | | 0.14263626 | 0.348572118 | | | ENSSSCG00000030522 |
| **KEGG pathways (top 15) of the downregulated genes in entire males compared to immunocastrated pigs** | | | | | | | | | |
| AMPK signaling pathway | ssc04152 | | | | 5.8449E-05 | 0.001402766 | | | ENSSSCG00000017933, GYS2, PCK1, SCD |
| Pentose phosphate pathway | ssc00030 | | | | 0.00085547 | 0.010265605 | | | PGD, G6PD |
| Insulin signaling pathway | ssc04910 | | | | 0.00168225 | 0.013458033 | | | ENSSSCG00000017933, GYS2, PCK1 |
| Glutathione metabolism | ssc00480 | | | | 0.00367118 | 0.022027051 | | | PGD, G6PD |
| PPAR signaling pathway | ssc03320 | | | | 0.0065562 | 0.027695537 | | | PCK1, SCD |
| Adipocytokine signaling pathway | ssc04920 | | | | 0.00692388 | 0.027695537 | | | PCK1, ENSSSCG00000017933 |
| Carbon metabolism | ssc01200 | | | | 0.01325506 | 0.045445922 | | | PGD, G6PD |
| FoxO signaling pathway | ssc04068 | | | | 0.02288203 | 0.068646081 | | | PCK1, ENSSSCG00000017933 |
| Nitrogen metabolism | ssc00910 | | | | 0.02736098 | 0.07296262 | | | CA3 |
| Biosynthesis of unsaturated fatty acids | ssc01040 | | | | 0.03072935 | 0.073750449 | | | SCD |
| Renin-angiotensin system | ssc04614 | | | | 0.0357606 | 0.078023118 | | | ACE2 |
| Proximal tubule bicarbonate reclamation | ssc04964 | | | | 0.0391006 | 0.078201202 | | | PCK1 |
| Metabolic pathways | ssc01100 | | | | 0.0502981 | 0.089739741 | | | ENSSSCG00000025108, PGD, PCK1, CKB, G6PD |
| Citrate cycle (TCA cycle) | ssc00020 | | | | 0.05234818 | 0.089739741 | | | PCK1 |
| Pyruvate metabolism | ssc00620 | | | | 0.06379361 | 0.102069775 | | | PCK1 |
| **KEGG enrichment analysis of the upregulated genes in immunocastrated compared to surgically castrated pigs** | | | | | | | | | |
| Retinol metabolism | | ssc00830 | | | 0.03056099 | | 0.130702654 | | ENSSSCG00000024919 |
| Legionellosis | | ssc05134 | | | 0.03602403 | | 0.130702654 | | HSPA1L |
| Antigen processing and presentation | | ssc04612 | | | 0.03820127 | | 0.130702654 | | HSPA1L |
| Estrogen signaling pathway | | ssc04915 | | | 0.05385107 | | 0.130702654 | | HSPA1L |
| Toxoplasmosis | | ssc05145 | | | 0.06344615 | | 0.130702654 | | HSPA1L |
| Spliceosome | | ssc03040 | | | 0.07084681 | | 0.130702654 | | HSPA1L |
| Measles | | ssc05162 | | | 0.07295134 | | 0.130702654 | | HSPA1L |
| Protein processing in endoplasmic reticulum | | ssc04141 | | | 0.08807814 | | 0.130702654 | | HSPA1L |
| Influenza A | | ssc05164 | | | 0.09117924 | | 0.130702654 | | HSPA1L |
| Epstein-Barr virus infection | | ssc05169 | | | 0.10195658 | | 0.130702654 | | HSPA1L |
| Endocytosis | | ssc04144 | | | 0.11059455 | | 0.130702654 | | HSPA1L |
| MAPK signaling pathway | | ssc04010 | | | 0.13210237 | | 0.143110906 | | HSPA1L |
| Metabolic pathways | | ssc01100 | | | 0.49084519 | | 0.490845193 | | ENSSSCG00000024919 |
| **KEGG enrichment analysis of the downregulated genes in immunocastrated compared to surgically castrated pigs** | | | | | | | | | |
| Oxidative phosphorylation | | ssc00190 | | | 0.02321284 | | 0.043629745 | | ND6 |
| Parkinson's disease | | ssc05012 | | | 0.02610702 | | 0.043629745 | | ND6 |
| Huntington's disease | | ssc05016 | | | 0.03272231 | | 0.043629745 | | TGM2 |
| Metabolic pathways | | ssc01100 | | | 0.1875776 | | 0.187577602 | | ND6 |
| **KEGG enrichment analysis of the upregulated genes in entire males compared to surgically castrated pigs** | | | | | | | | | |
| Protein processing in endoplasmic reticulum | | | ssc04141 | 0.00080471 | | | | 0.02150356 | HSPH1, DNAJB1, HSPA8, HSPA1L |
| Protein digestion and absorption | | | ssc04974 | 0.00119464 | | | | 0.02150356 | COL12A1, COL1A2, COL6A3 |
| Influenza A | | | ssc05164 | 0.00911319 | | | | 0.09162525 | DNAJB1, HSPA8, HSPA1L |
| Legionellosis | | | ssc05134 | 0.01197491 | | | | 0.09162525 | HSPA8, HSPA1L |
| Antigen processing and presentation | | | ssc04612 | 0.01339633 | | | | 0.09162525 | HSPA8, HSPA1L |
| Complement and coagulation cascades | | | ssc04610 | 0.01527088 | | | | 0.09162525 | C7, F3 |
| ECM-receptor interaction | | | ssc04512 | 0.01891012 | | | | 0.09725203 | COL1A2, COL6A3 |
| MAPK signaling pathway | | | ssc04010 | 0.02545131 | | | | 0.10277567 | ENSSSCG00000009585, HSPA8, HSPA1L |
| Estrogen signaling pathway | | | ssc04915 | 0.02569392 | | | | 0.10277567 | HSPA8, HSPA1L |
| Toxoplasmosis | | | ssc05145 | 0.03493083 | | | | 0.12575098 | HSPA8, HSPA1L |
| Spliceosome | | | ssc03040 | 0.04287069 | | | | 0.13575691 | HSPA8, HSPA1L |
| Measles | | | ssc05162 | 0.0452523 | | | | 0.13575691 | HSPA8, HSPA1L |
| Tyrosine metabolism | | | ssc00350 | 0.08192931 | | | | 0.19547404 | ENSSSCG00000030522 |
| Epstein-Barr virus infection | | | ssc05169 | 0.0831368 | | | | 0.19547404 | HSPA8, HSPA1L |
| Prion diseases | | | ssc05020 | 0.08423632 | | | | 0.19547404 | C7 |
| **KEGG enrichment analysis of the downregulated genes in entire males compared to surgically castrated pigs** | | | | | | | | | |
| Proximal tubule bicarbonate reclamation | | | ssc04964 | 0.0158358 | | | | 0.11001186 | PCK1 |
| Citrate cycle (TCA cycle) | | | ssc00020 | 0.02128857 | | | | 0.11001186 | PCK1 |
| Pyruvate metabolism | | | ssc00620 | 0.0260365 | | | | 0.11001186 | PCK1 |
| Glycolysis / Gluconeogenesis | | | ssc00010 | 0.03747745 | | | | 0.11001186 | PCK1 |
| Legionellosis | | | ssc05134 | 0.04414843 | | | | 0.11001186 | CXCL2 |
| PPAR signaling pathway | | | ssc03320 | 0.04680467 | | | | 0.11001186 | PCK1 |
| Adipocytokine signaling pathway | | | ssc04920 | 0.04813019 | | | | 0.11001186 | PCK1 |
| Salmonella infection | | | ssc05132 | 0.05538972 | | | | 0.11077945 | CXCL2 |
| TNF signaling pathway | | | ssc04668 | 0.07363502 | | | | 0.11868046 | CXCL2 |
| AMPK signaling pathway | | | ssc04152 | 0.07942905 | | | | 0.11868046 | PCK1 |
| FoxO signaling pathway | | | ssc04068 | 0.08901035 | | | | 0.11868046 | PCK1 |
| Insulin signaling pathway | | | ssc04910 | 0.08901035 | | | | 0.11868046 | PCK1 |
| Chemokine signaling pathway | | | ssc04062 | 0.11410542 | | | | 0.14043744 | CXCL2 |
| Huntington's disease | | | ssc05016 | 0.12457332 | | | | 0.1423695 | CXCL2 |
| PI3K-Akt signaling pathway | | | ssc04151 | 0.20535748 | | | | 0.21904797 | PCK1 |
