# Supplementary material for: Adipose Tissue Gene Expression of Entire Male, Immunocastrated and Surgically Castrated Pigs
Source: Int J Mol Sci. 2021 Feb 10;22(4):1768. doi: 10.3390/ijms22041768 (PMC7916650; doi:10.3390/ijms22041768)
Supplement: Supplementary file 1 [file ijms-22-01768-s001.zip › Supplementary_Figure_S1.docx]

**Supplementary Figure S1: Co-expression Venn diagram of the expressed genes between entire males, immunocastrated pigs and surgically castrated pigs.**


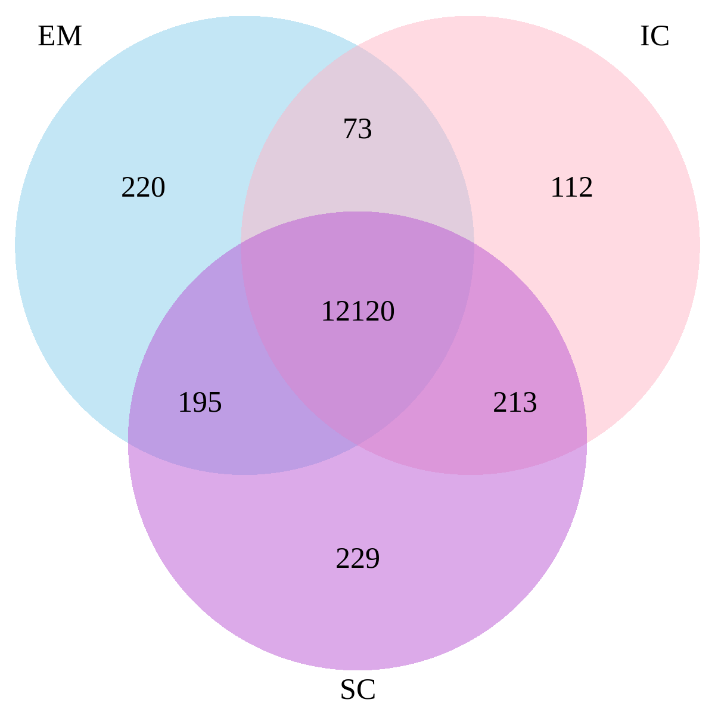


**Supplementary Figure S1.** Co-expression Venn diagram of the expressed genes between entire males (EM), immunocastrated pigs (IC) and surgically castrated pigs (SC).
