# Supplementary material for: Adipose Tissue Gene Expression of Entire Male, Immunocastrated and Surgically Castrated Pigs
Source: Int J Mol Sci. 2021 Feb 10;22(4):1768. doi: 10.3390/ijms22041768 (PMC7916650; doi:10.3390/ijms22041768)
Supplement: Supplementary file 1 [file ijms-22-01768-s001.zip › Supplementary_Figure_S2.docx]

**Supplementary Figure S2: Validation of differentially expressed genes by qPCR (12 individual animals per group) in comparison to RNA-seq (RNA pool of 12 animals per group).**


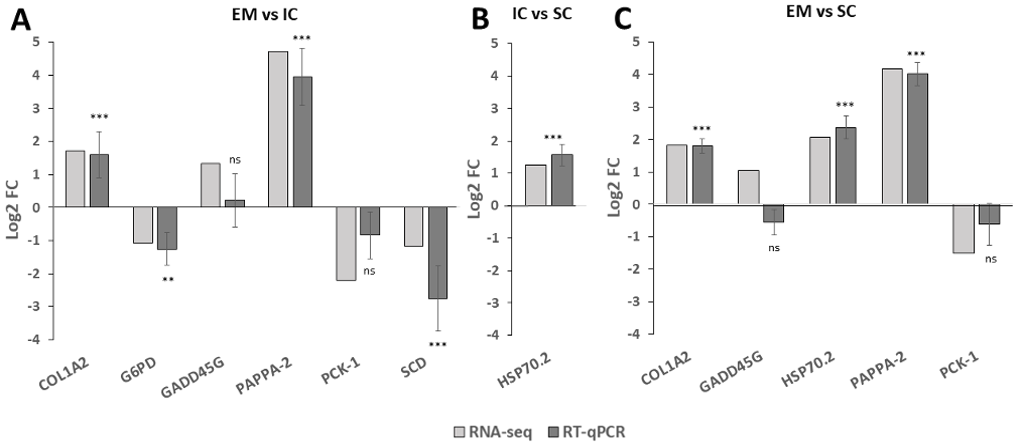


**Supplementary Figure S2.** Validation of differentially expressed genes by qPCR (12 individual animals per group) in comparison to RNA-seq (RNA pool of 12 animals per group). Log_2_ fold change indicates A) the expression difference between entire males (EM) compared to immunocastrated pigs (IC) with IC as a control; B) between IC compared to surgical castrates (SC) with SC as a control; C) between EM and SC with SC as a control. Log2 FC = log_2_ fold change; COL1A2 = collagen, type I, alpha 2; G6PD = glucose-6-phosphate dehydrogenase; GADD45G = growth arrest and DNA damage inducible, gamma; HSP70.2 = heat shock 70 KDa protein 1A/1B; PAPPA-2 = pappalysin-2; PCK1 = phosphoenolpyruvate carboxykinase 1; SCD = stearoyl CoA desaturase. In the case of statistical significance (reported as Tukey adjusted P-value), the asterisks are drawn (*** P < 0.001; ** P < 0.01; * P < 0.05; ns = not significant).
