# Supplementary material for: Adipose Tissue Gene Expression of Entire Male, Immunocastrated and Surgically Castrated Pigs
Source: Int J Mol Sci. 2021 Feb 10;22(4):1768. doi: 10.3390/ijms22041768 (PMC7916650; doi:10.3390/ijms22041768)
Supplement: Supplementary file 1 [file ijms-22-01768-s001.zip › Supplementary_Table_S1.docx]

**Supplementary Table S1: Summary of data quality control.**

**Supplementary Table S1.** Summary of data quality control

| **Sample name** | **Raw reads** | **Clean reads** | **Error rate (%)** | **Q20 (%)** | **Q30 (%)** | **GC content (%)** |
| --- | --- | --- | --- | --- | --- | --- |
| EM pool | 77929748 | 77096968 | 0.03 | 96.76 | 91.7 | 50.08 |
| IC pool | 98202924 | 96664708 | 0.03 | 97.42 | 93.32 | 50.12 |
| SC pool | 92447540 | 91031292 | 0.03 | 97.44 | 93.36 | 50.19 |

EM = entire males; IC = immunocastated pigs; SC = surgically castrated pigs.
