# Supplementary material for: Adipose Tissue Gene Expression of Entire Male, Immunocastrated and Surgically Castrated Pigs
Source: Int J Mol Sci. 2021 Feb 10;22(4):1768. doi: 10.3390/ijms22041768 (PMC7916650; doi:10.3390/ijms22041768)
Supplement: Supplementary file 1 [file ijms-22-01768-s001.zip › Supplementary_Table_S2.docx]

**Supplementary Table S2: Overview of the mapping status.**

**Supplementary Table S2.** Overview of the mapping status

|  | **EM pool** | **IC pool** | **SC pool** |
| --- | --- | --- | --- |
| Total number of filtered reads | 77096968 | 96664708 | 91031292 |
| Number of reads mapped to reference genome | 59159713 (76.73%) | 74573819 (77.15%) | 70394914 (77.33%) |
| Multiple mapped reads | 3492570 (4.53%) | 4099587 (4.24%) | 3915451 (4.3%) |
| Uniquely mapped reads | 55667143 (72.2%) | 70474232 (72.91%) | 66479463 (73.03%) |
| Reads mapped to positive strand | 27808461 (36.07%) | 35172769 (36.39%) | 33207720 (36.48%) |
| Reads mapped to negative strand | 27858682 (36.13%) | 35301463 (36.52%) | 33271743 (36.55%) |
| Non-spliced reads | 35027984 (45.43%) | 45197350 (46.76%) | 42301161 (46.47%) |
| Splice reads | 20639159 (26.77%) | 25276882 (26.15%) | 24178302 (26.56%) |

EM = entire males; IC = immunocastated pigs; SC = surgically castrated pigs.
