# Supplementary material for: Adipose Tissue Gene Expression of Entire Male, Immunocastrated and Surgically Castrated Pigs
Source: Int J Mol Sci. 2021 Feb 10;22(4):1768. doi: 10.3390/ijms22041768 (PMC7916650; doi:10.3390/ijms22041768)
Supplement: Supplementary file 1 [file ijms-22-01768-s001.zip › Supplementary_Table_S3.docx]

**Supplementary Table S3: Differentially expressed genes between entire males and immunocastrated pigs, between immunocastrated and surgically castrated pigs and between entire males and surgically castrated pigs.**

**Supplementary Table S3**. Differentially expressed genes between entire males and immunocastrated pigs, between immunocastrated and surgically castrated pigs and between entire males and surgically castrated pigs.

| **Gene name** | | **Gene description** | | **Log_2_ Fold change** | | | | **P-value** | | | **Q-value** |  |
| --- | --- | --- | --- | --- | --- | --- | --- | --- | --- | --- | --- | --- |
| **Upregulated genes in entire males compared to immunocastrated pigs** | | | | | | | | | | | |  |
| ENSSSCG00000018063 | | / | | 5.7817 | | | | 2.348E-179 | | | 3.978E-176 |  |
| ENSSSCG00000018061 | | / | | 5.3196 | | | | 9.0414E-60 | | | 7.2925E-57 |  |
| PAPPA2 | | pappalysin 2 | | 4.7193 | | | | 9.341E-15 | | | 2.0818E-12 |  |
| ENSSSCG00000025162 | | / | | 3.7856 | | | | 2.0538E-18 | | | 5.7029E-16 |  |
| RRP12 | | ribosomal RNA processing 12 | | 3.3609 | | | | 3.3893E-07 | | | 2.8479E-05 |  |
| ENSSSCG00000022000 | | / | | 3.2368 | | | | 2.7032E-07 | | | 2.3009E-05 |  |
| P4HA3 | | prolyl 4-hydroxylase subunit alpha 3 | | 3.0523 | | | | 3.6874E-06 | | | 0.00026577 |  |
| C7 | | complement component C7 precursor | | 2.0411 | | | | 4.5466E-05 | | | 0.0026833 |  |
| ENSSSCG00000021015 | | / | | 1.9717 | | | | 8.8292E-87 | | | 8.3083E-84 |  |
| ENSSSCG00000017579 | | / | | 1.9633 | | | | 1.077E-107 | | | 1.216E-104 |  |
| TNFSF9 | | TNF superfamily member 9 | | 1.912 | | | | 3.6335E-05 | | | 0.0021747 |  |
| RRP1B | | ribosomal RNA processing 1B | | 1.8429 | | | | 2.4225E-05 | | | 0.0014975 |  |
| CHAC1 | | ChaC glutathione specific gamma-glutamylcyclotransferase 1 | | 1.8334 | | | | 1.952E-07 | | | 1.7131E-05 |  |
| ENSSSCG00000013393 | | / | | 1.8125 | | | | 1.3751E-11 | | | 2.2834E-09 |  |
| ENSSSCG00000029074 | | / | | 1.7982 | | | | 5.358E-272 | | | 1.297E-268 |  |
| RRP9 | | ribosomal RNA processing 9 | | 1.7183 | | | | 1.7793E-09 | | | 2.1998E-07 |  |
| COL1A2 | | - | | 1.7095 | | | | 0 | | | 0 |  |
| PPL | | periplakin | | 1.7073 | | | | 9.6726E-22 | | | 3.2767E-19 |  |
| POSTN | | periostin | | 1.6377 | | | | 7.624E-236 | | | 1.614E-232 |  |
| DCN | | decorin | | 1.6262 | | | | 2.676E-288 | | | 7.554E-285 |  |
| SCARA5 | | scavenger receptor class A member 5 | | 1.5614 | | | | 4.7186E-11 | | | 7.4003E-09 |  |
| ENSSSCG00000027593 | | / | | 1.5351 | | | | 1.083E-15 | | | 2.6975E-13 |  |
| ENSSSCG00000021943 | | / | | 1.5318 | | | | 2.8475E-79 | | | 2.4116E-76 |  |
| MFAP2 | | microfibrillar associated protein 2 | | 1.4946 | | | | 2.5909E-09 | | | 3.1572E-07 |  |
| IL1RAP | | interleukin 1 receptor accessory protein | | 1.4074 | | | | 8.2461E-05 | | | 0.0045794 |  |
| ENSSSCG00000017581 | | / | | 1.3834 | | | | 1.1426E-97 | | | 1.2096E-94 |  |
| ENSSSCG00000014118 | | / | | 1.3792 | | | | 6.6968E-05 | | | 0.0037937 |  |
| RCAN1 | | regulator of calcineurin 1 | | 1.359 | | | | 1.7139E-09 | | | 2.1477E-07 |  |
| GADD45G | | growth arrest and DNA damage inducible gamma | | 1.3379 | | | | 3.9269E-21 | | | 1.3042E-18 |  |
| ENSSSCG00000008898 | | / | | 1.3305 | | | | 5.4584E-05 | | | 0.0031771 |  |
| CRISPLD2 | | cysteine rich secretory protein LCCL domain containing 2 | | 1.318 | | | | 2.4214E-05 | | | 0.0014975 |  |
| OMD | | osteomodulin | | 1.304 | | | | 1.119E-12 | | | 2.1785E-10 |  |
| COL6A3 | | collagen type VI alpha 3 chain | | 1.304 | | | | 5.341E-171 | | | 8.223E-168 |  |
| LGALS3 | | galectin 3 | | 1.286 | | | | 1.4479E-08 | | | 1.5522E-06 |  |
| C1QTNF6 | | C1q and tumor necrosis factor related protein 6 | | 1.2841 | | | | 1.6622E-22 | | | 5.8656E-20 |  |
| ANPEP | | alanyl aminopeptidase | | 1.2636 | | | | 4.9523E-15 | | | 1.1491E-12 |  |
| GLT8D2 | | glycosyltransferase 8 domain containing 2 | | 1.2505 | | | | 9.3878E-06 | | | 0.000631 |  |
| ENSSSCG00000004484 | | / | | 1.2349 | | | | 1.9619E-10 | | | 2.7924E-08 |  |
| PLAC9 | | placenta specific 9 | | 1.2298 | | | | 1.7016E-11 | | | 2.7983E-09 |  |
| FMOD | | fibromodulin | | 1.2198 | | | | 1.4444E-08 | | | 1.5522E-06 |  |
| ASPN | | asporin | | 1.2131 | | | | 1.9678E-12 | | | 3.7035E-10 |  |
| ENSSSCG00000030522 | | / | | 1.2088 | | | | 5.4193E-08 | | | 5.4638E-06 |  |
| ANGPTL1 | | angiopoietin like 1 | | 1.1868 | | | | 3.8639E-05 | | | 0.0022963 |  |
| C4BPA | | - | | 1.1864 | | | | 2.7249E-05 | | | 0.0016784 |  |
| ENSSSCG00000003603 | | / | | 1.1804 | | | | 7.5074E-10 | | | 9.8574E-08 |  |
| MMP2 | | 72 kDa type IV collagenase precursor | | 1.139 | | | | 3.718E-119 | | | 4.498E-116 |  |
| ITGBL1 | | integrin subunit beta like 1 | | 1.1082 | | | | 3.736E-07 | | | 3.1173E-05 |  |
| C1QTNF1 | | C1q and TNF related 1 | | 1.1081 | | | | 1.4728E-06 | | | 0.00011288 |  |
| CYP2B6 | | cytochrome P450 family 2 subfamily B member 6 | | 1.0973 | | | | 5.083E-11 | | | 7.827E-09 |  |
| AGTR1 | | angiotensin II receptor type 1 | | 1.0904 | | | | 2.8236E-09 | | | 3.3919E-07 |  |
| CPXM1 | | carboxypeptidase X | | 1.0883 | | | | 2.7573E-11 | | | 4.4906E-09 |  |
| AEBP1 | | AE binding protein 1 | | 1.0745 | | | | 7.4769E-43 | | | 4.523E-40 |  |
| MMP27 | | matrix metallopeptidase 27 | | 1.0637 | | | | 8.4072E-05 | | | 0.0046536 |  |
| PDGFRL | | platelet derived growth factor receptor like | | 1.0583 | | | | 5.5385E-05 | | | 0.0032017 |  |
| P4HA2 | | prolyl 4-hydroxylase subunit alpha | | 1.057 | | | | 1.0157E-07 | | | 9.2495E-06 |  |
| ENSSSCG00000022506 | | / | | 1.0515 | | | | 2.1156E-27 | | | 9.1881E-25 |  |
| S100A6 | | S100 calcium binding protein A6 | | 1.0245 | | | | 1.1641E-11 | | | 1.9523E-09 |  |
| COL14A1 | | collagen type XIV alpha 1 chain | | 1.0204 | | | | 6.2196E-25 | | | 2.6337E-22 |  |
| TMEM47 | | transmembrane protein 47 | | 1.0204 | | | | 6.6516E-08 | | | 6.5125E-06 |  |
| RCN1 | | reticulocalbin 1 | | 1.0154 | | | | 4.8535E-20 | | | 1.5511E-17 |  |
| **Downregulated genes in entire males compared to immunocastrated pigs** | | | | | | | | | | | |  |
| PGD | | phosphogluconate dehydrogenase | | -1.0053 | | | | 2.3813E-23 | | | 8.963E-21 |  |
| ENSSSCG00000021159 | | / | | -1.0343 | | | | 8.7431E-05 | | | 0.0048238 |  |
| ENSSSCG00000021220 | | / | | -1.0549 | | | | 2.4021E-07 | | | 2.0759E-05 |  |
| G6PD | | glucose-6-phosphate dehydrogenase | | -1.0728 | | | | 2.5444E-13 | | | 5.1306E-11 |  |
| ENSSSCG00000017933 | | / | | -1.0932 | | | | 5.6866E-07 | | | 4.6307E-05 |  |
| SLPI | | antileukoproteinase | | -1.0942 | | | | 2.2209E-14 | | | 4.8228E-12 |  |
| UCP3 | | uncoupling protein 3 | | -1.1511 | | | | 4.886E-14 | | | 1.0345E-11 |  |
| SCD | | stearoyl-CoA desaturase (delta-9-desaturase) | | -1.157 | | | | 0 | | | 0 |  |
| CLSTN3 | | calsyntenin 3 | | -1.1941 | | | | 4.3698E-09 | | | 5.0968E-07 |  |
| CA3 | | Carbonic anhydrase 3 | | -1.298 | | | | 2.6778E-84 | | | 2.3872E-81 |  |
| ENSSSCG00000023177 | | / | | -1.3131 | | | | 0 | | | 0 |  |
| SLC25A34 | | solute carrier family 25 member 34 | | -1.3376 | | | | 4.7831E-13 | | | 9.5313E-11 |  |
| GYS2 | | glycogen synthase 2 | | -1.3444 | | | | 3.0343E-05 | | | 0.0018488 |  |
| RDH5 | | retinol dehydrogenase 5 | | -1.353 | | | | 2.7894E-11 | | | 4.4997E-09 |  |
| ACE2 | | angiotensin I converting enzyme 2 | | -1.477 | | | | 8.5223E-36 | | | 4.3743E-33 |  |
| TTC25 | | tetratricopeptide repeat domain 25 | | -1.6725 | | | | 1.1018E-07 | | | 9.8223E-06 |  |
| ENSSSCG00000028753 | | / | | -1.7177 | | | | 3.2014E-07 | | | 2.7113E-05 |  |
| AZGP1 | | alpha-2-glycoprotein 1 | | -1.7818 | | | | 1.9692E-06 | | | 0.00014693 |  |
| RBP1 | | retinol binding protein 1 | | -1.8291 | | | | 1.244E-09 | | | 1.6084E-07 |  |
| ENSSSCG00000002824 | | / | | -1.8298 | | | | 3.2073E-05 | | | 0.0019402 |  |
| PCK1 | | phosphoenolpyruvate carboxykinase 1 | | -2.1885 | | | | 4.0627E-50 | | | 2.8673E-47 |  |
| IGLC | | - | | -2.3957 | | | | 1.1015E-08 | | | 1.2194E-06 |  |
| IGKC | | immunoglobulin kappa constant | | -2.6426 | | | | 6.8254E-05 | | | 0.0038536 |  |
| **Upregulated genes in immunocastrated pigs compared to surgically castrated pigs** | | | | | | | | | | | | |
| ENSSSCG00000002824 | / | | | | 2.5337 | | 4.2087E-07 | | | 0.00011092 | | |
| AZGP1 | alpha-2-glycoprotein 1 | | | | 2.1509 | | 1.1578E-07 | | | 3.4197E-05 | | |
| IGLC | / | | | | 1.6043 | | 2.8116E-05 | | | 0.0048653 | | |
| RBP1 | retinol binding protein 1 | | | | 1.3207 | | 6.1988E-06 | | | 0.0012705 | | |
| HSP70.2 | Sus scrofa heat shock protein 70.2 (HSP70.2) | | | | 1.2511 | | 5.395E-131 | | | 2.31E-127 | | |
| RDH16 | / | | | | 1.0686 | | 9.1188E-13 | | | 4.5945E-10 | | |
| **Downregulated genes in immunocastrated pigs compared to surgically castrated pigs** | | | | | | | | | | | | |
| TGM2 | transglutaminase 2 | | | | -1.0152 | | 2.1187E-17 | | | 1.2098E-14 | | |
| ND6 | NADH-ubiquinone oxidoreductase chain 6 | | | | -1.2384 | | 1.5701E-83 | | | 3.8425E-80 | | |
| RRP9 | ribosomal RNA processing 9 | | | | -1.8245 | | 8.6476E-12 | | | 4.0038E-09 | | |
| RRP1B | ribosomal RNA processing 1B | | | | -1.9726 | | 1.3265E-06 | | | 0.0003113 | | |
| PTX3 | pentraxin 3 | | | | -2.0656 | | 2.1791E-27 | | | 1.7776E-24 | | |
| ENSSSCG00000025162 | / | | | | -3.1131 | | 2.7874E-11 | | | 1.2244E-08 | | |
| RRP12 | ribosomal RNA processing 12 homolog | | | | -3.5869 | | 5.2316E-09 | | | 1.829E-06 | | |
| ENSSSCG00000018061 | / | | | | -5.4304 | | 7.5666E-68 | | | 1.4403E-64 | | |
| ENSSSCG00000018063 | / | | | | -5.7611 | | 8.642E-186 | | | 7.402E-182 | | |
|  |  | | | |  | |  | | |  | | |
| **Upregulated genes in entire males compared to surgically castrated pigs** | | | | | | | | | | | | |
| PAPPA2 | | | pappalysin 2 | | | 4.1662 | | | 7.6244E-14 | | 1.9592E-11 | |
| ENSSSCG00000022000 | | | / | | | 2.9311 | | | 1.4638E-06 | | 0.00014518 | |
| P4HA3 | | | prolyl 4-hydroxylase subunit alpha 3 | | | 2.619 | | | 2.8885E-05 | | 0.0021677 | |
| ENSSSCG00000030522 | | | / | | | 2.5643 | | | 4.8952E-18 | | 1.6279E-15 | |
| C7 | | | complement component C7 precursor | | | 2.2787 | | | 2.5343E-05 | | 0.0019449 | |
| HSP70.2 | | | Sus scrofa heat shock protein 70.2 (HSP70.2) | | | 2.0804 | | | 0 | | 0 | |
| ENSSSCG00000013393 | | | / | | | 1.9859 | | | 6.2458E-12 | | 1.3078E-09 | |
| ENSSSCG00000017579 | | | / | | | 1.8511 | | | 2.7067E-91 | | 4.1732E-88 | |
| COL1A2 | | | / | | | 1.8217 | | | 0 | | 0 | |
| POSTN | | | periostin | | | 1.7615 | | | 2.566E-235 | | 7.253E-232 | |
| ENSSSCG00000021015 | | | / | | | 1.7221 | | | 4.38E-66 | | 5.306E-63 | |
| ENSSSCG00000027593 | | | / | | | 1.7187 | | | 1.9935E-16 | | 6.1473E-14 | |
| ENSSSCG00000029074 | | | / | | | 1.6404 | | | 9.44E-216 | | 2.287E-212 | |
| ENSSSCG00000013784 | | | / | | | 1.5993 | | | 5.3574E-35 | | 3.7859E-32 | |
| ENSSSCG00000021943 | | | / | | | 1.5879 | | | 2.5273E-74 | | 3.5719E-71 | |
| DCN | | | decorin | | | 1.5389 | | | 4.406E-237 | | 1.494E-233 | |
| 5_8S_rRNA | | | 5.8S ribosomal RNA | | | 1.5106 | | | 1.5199E-06 | | 0.00014815 | |
| CHAC1 | | | ChaC glutathione specific gamma-glutamylcyclotransferase 1 | | | 1.5023 | | | 1.9481E-05 | | 0.0015585 | |
| C1QTNF6 | | | C1q and tumor necrosis factor related protein 6 | | | 1.4717 | | | 5.6753E-24 | | 2.9167E-21 | |
| ENSSSCG00000017581 | | | / | | | 1.4508 | | | 8.2003E-92 | | 1.3908E-88 | |
| OMD | | | osteomodulin | | | 1.3005 | | | 4.6273E-11 | | 9.3428E-09 | |
| F3 | | | coagulation factor III | | | 1.2923 | | | 6.2201E-60 | | 6.5933E-57 | |
| PPL | | | periplakin | | | 1.2727 | | | 5.8741E-13 | | 1.3283E-10 | |
| C1QTNF1 | | | C1q and TNF related 1 | | | 1.2646 | | | 8.9798E-07 | | 9.1064E-05 | |
| S100A6 | | | S100 calcium binding protein A6 | | | 1.2352 | | | 5.7485E-13 | | 1.3175E-10 | |
| SCARA5 | | | scavenger receptor class A member | | | 1.2161 | | | 4.1908E-07 | | 4.6153E-05 | |
| RCAN1 | | | regulator of calcineurin 1 | | | 1.2021 | | | 4.1748E-07 | | 4.6153E-05 | |
| ENSSSCG00000029781 | | | / | | | 1.1691 | | | 1.6989E-07 | | 2.0729E-05 | |
| RCN1 | | | reticulocalbin 1 | | | 1.155 | | | 2.0931E-20 | | 8.2557E-18 | |
| AEBP1 | | | AE binding protein 1 | | | 1.104 | | | 2.7472E-37 | | 2.1178E-34 | |
| HSPA1L | | | heat shock protein family A (Hsp70) member 1 like | | | 1.0978 | | | 4.0723E-06 | | 0.00037536 | |
| PLAC9 | | | placenta specific 9 | | | 1.0968 | | | 1.8997E-08 | | 2.8768E-06 | |
| COL6A3 | | | collagen type VI alpha 3 chain | | | 1.0733 | | | 3.836E-106 | | 7.228E-103 | |
| ENSSSCG00000023594 | | | / | | | 1.0635 | | | 7.0814E-05 | | 0.0048987 | |
| GADD45G | | | growth arrest and DNA damage inducible gamma | | | 1.0461 | | | 1.2071E-12 | | 2.6587E-10 | |
| HSPH1 | | | heat shock protein 105 kDa | | | 1.0427 | | | 6.3278E-08 | | 8.1303E-06 | |
| RCN3 | | | reticulocalbin 3 | | | 1.028 | | | 9.4197E-08 | | 1.1922E-05 | |
| ASPN | | | asporin | | | 1.0123 | | | 3.1947E-08 | | 4.4411E-06 | |
| ENSSSCG00000004484 | | | / | | | 1.0109 | | | 8.2246E-07 | | 0.00008403 | |
| SERPINF1 | | | serpin family F member 1 | | | 1.0038 | | | 8.9274E-19 | | 3.2215E-16 | |
| **Downregulated genes in entire males compared to surgically castrated pigs** | | | | | | | | | | | | |
| CXCL2 | | | C-X-C motif chemokine 2 precursor | | | -1.0274 | | | 1.5826E-07 | | 0.00001945 | |
| SLC25A34 | | | solute carrier family 25 member 34 | | | -1.0454 | | | 9.5221E-10 | | 1.6649E-07 | |
| UCP3 | | | uncoupling protein 3 | | | -1.059 | | | 5.295E-15 | | 1.5221E-12 | |
| ENSSSCG00000002825 | | | / | | | -1.0719 | | | 2.8859E-18 | | 9.9888E-16 | |
| MYOC | | | myocilin | | | -1.3611 | | | 7.058E-73 | | 9.208E-70 | |
| TGM2 | | | transglutaminase 2 | | | -1.4827 | | | 8.9516E-27 | | 5.0607E-24 | |
| PCK1 | | | phosphoenolpyruvate carboxykinase 1 | | | -1.4995 | | | 2.8363E-23 | | 1.3362E-20 | |
| PTX3 | | | pentraxin 3 | | | -2.1512 | | | 1.016E-27 | | 5.942E-25 | |
