# Supplementary material for: Adipose Tissue Gene Expression of Entire Male, Immunocastrated and Surgically Castrated Pigs
Source: Int J Mol Sci. 2021 Feb 10;22(4):1768. doi: 10.3390/ijms22041768 (PMC7916650; doi:10.3390/ijms22041768)
Supplement: Supplementary file 1 [file ijms-22-01768-s001.zip › Supplementary_Table_S4.docx]

**Supplementary Table S4: Gene ontology enrichment analysis of the upregulated and downregulated genes between entire males and immunocastrated pigs, between immunocastrated and surgically castrated pigs and between entire males and surgically castrated pigs.**

**Supplementary Table S4.** Gene ontology (GO) enrichment analysis of the upregulated and downregulated genes between entire males and immunocastrated pigs, between immunocastrated and surgically castrated pigs and between entire males and surgically castrated pigs.

| **GO accession** | **Description** | **Term type** | | | **Over represented *p*-value** | | **Corrected *p*-value** | | **Gene names** |
| --- | --- | --- | --- | --- | --- | --- | --- | --- | --- |
| **GO enrichment analysis (top 15 terms) of the upregulated genes in entire males compared to immunocastrated pigs** | | | | | | | | | |
| GO:0031012 | extracellular matrix | cellular component | | | 1.4004E-14 | | 2.558E-10 | | POSTN, MMP2, CRISPLD2, SPON1, MMP27, DCN, OMD, ASPN, LGALS3, COL14A1, COL6A3, AEBP1, MFAP2, FMOD |
| GO:0005615 | extracellular space | cellular component | | | 1.4004E-10 | | 0.000001279 | | ANPEP, AEBP1, COL6A3, DCN, OMD, ITGBL1, IL1RAP, ANGPTL1, POSTN, MMP2, C1QTNF6, FMOD, COL14A1, LGALS3, ENSSSCG00000021379, CPXM1 |
| GO:0005576 | extracellular region | cellular component | | | 4.9794E-09 | | 0.000028412 | | OMD, ENSSSCG00000014118, DCN, COL6A3, AEBP1, ANPEP, POSTN, MMP2, CRISPLD2, ANGPTL1, IL1RAP, ITGBL1, PPL, ASPN, S100A6, LGALS3, COL14A1, FMOD, PAPPA2, MFAP2, C1QTNF6 , CPXM1, C7, ENSSSCG00000021379 |
| GO:0044421 | extracellular region part | cellular component | | | 6.2218E-09 | | 0.000028412 | | POSTN, MMP2, CRISPLD2, ANGPTL1, IL1RAP, PPL, ITGBL1, OMD, DCN, AEBP1, COL6A3, ANPEP, CPXM1, C7, ENSSSCG00000021379, ASPN, S100A6, LGALS3, COL14A1, PAPPA2, FMOD, MFAP2, C1QTNF6 |
| GO:0005578 | proteinaceous extracellular matrix | cellular component | | | 3.9177E-06 | | 0.014312 | | COL6A3, MFAP2, FMOD, POSTN, OMD, ASPN, CRISPLD2 |
| GO:0005201 | extracellular matrix structural constituent | molecular function | | | 0.000011031 | | 0.033581 | | ENSSSCG00000021943, ENSSSCG00000022000, COL1A2, ENSSSCG00000017581 |
| GO:0070206 | protein trimerization | biological process | | | 0.000013911 | | 0.036301 | | ENSSSCG00000009672, C1QTNF6, ENSSSCG00000021379 |
| GO:0004656 | procollagen-proline 4-dioxygenase activity | molecular function | | | 0.000026769 | | 0.061119 | | P4HA3, P4HA2 |
| GO:0070208 | protein heterotrimerization | biological process | | | 0.0000412 | | 0.083618 | | C1QTNF6, ENSSSCG00000021379 |
| GO:0019798 | procollagen-proline dioxygenase activity | molecular function | | | 0.00013765 | | 0.25144 | | P4HA2, P4HA3 |
| GO:0008237 | metallopeptidase activity | molecular function | | | 0.00019667 | | 0.32659 | | MMP2, MMP27, AEBP1, PAPPA2, ANPEP |
| GO:0031545 | peptidyl-proline 4-dioxygenase activity | molecular function | | | 0.0002161 | | 0.32893 | | P4HA2, P4HA3 |
| GO:0005581 | collagen trimer | cellular component | | | 0.00028136 | | 0.34825 | | ENSSSCG00000017581, C1QTNF6, COL6A3 |
| GO:0005518 | collagen binding | molecular function | | | 0.00028138 | | 0.34825 | | ENSSSCG00000021379, DCN, ASPN |
| GO:0046914 | transition metal ion binding | molecular function | | | 0.00028599 | | 0.34825 | | MMP2, CPXM1, P4HA2, MMP27, S100A6, CYP2B22, ENSSSCG00000030522, P4HA3, AEBP1, ANPEP, PAPPA2 |
| **GO enrichment analysis (top 15 terms) of the downregulated genes in entire males compared to immunocastrated pigs** | | | | | | | | | |
| GO:0019322 | pentose biosynthetic process | biological process | | | 1.6373E-06 | | 0.025427 | | PGD, G6PD |
| GO:0016616 | oxidoreductase activity acting on the CH-OH group of donors NAD or NADP as acceptor | molecular function | | | 3.5066E-06 | | 0.025427 | | RDH5, PGD, ENSSSCG00000023044, G6PD |
| GO:0009051 | pentose-phosphate shunt oxidative branch | biological process | | | 0.000004918 | | 0.000004918 | | PGD, G6PD |
| GO:0016051 | carbohydrate biosynthetic process | biological process | | | 5.7276E-06 | | 0.025427 | | PGD, GYS2, PCK1, G6PD |
| GO:0016614 | oxidoreductase activity acting on CH-OH group of donors | molecular function | | | 6.9602E-06 | | 6.9602E-06 | | G6PD, RDH5, PGD, ENSSSCG00000023044 |
| GO:0044283 | small molecule biosynthetic process | biological process | | | 0.000018108 | | 0.044858 | | RBP1, G6PD, SCD, PGD, PCK1 |
| GO:0019321 | pentose metabolic process | biological process | | | 0.000020229 | | 0.044858 | | PGD, G6PD |
| GO:0046364 | monosaccharide biosynthetic process | biological process | | | 0.000020434 | | 0.044858 | | PCK1, PGD, G6PD |
| GO:0003823 | antigen binding | molecular function | | | 0.000024011 | | 0.044858 | | AZGP1, ENSSSCG00000008203, ENSSSCG00000010044 |
| GO:0032787 | monocarboxylic acid metabolic process | biological process | | | 0.000024558 | | 0.044858 | | RBP1, PGD, SCD, UCP3, PCK1 |
| GO:0044710 | single-organism metabolic process | biological process | | | 0.000029487 | | 0.048965 | | ENSSSCG00000008203, G6PD, ENSSSCG00000010044, RBP1, PCK1, RDH5, GYS2, UCP3, ENSSSCG00000023044, ENSSSCG00000028753, CA3, SCD, PGD |
| GO:0055114 | oxidation-reduction process | biological process | | | 0.000041904 | | 0.063784 | | RDH5, ENSSSCG00000023044, SCD, PGD, GYS2, ENSSSCG00000028753, G6PD |
| GO:0042571 | immunoglobulin complex circulating | cellular component | | | 0.000058449 | | 0.082125 | | ENSSSCG00000008203, ENSSSCG00000010044 |
| GO:0072562 | blood microparticle | cellular component | | | 0.000075083 | | 0.097962 | | ENSSSCG00000010044, ENSSSCG00000008203 |
| GO:0034987 | immunoglobulin receptor binding | molecular function | | | 0.000085326 | | 0.1039 | | ENSSSCG00000010044, ENSSSCG00000008203 |
| **GO enrichment analysis (top 15 terms) of the upregulated genes in immunocastrated compared to surgically castrated pigs** | | | | | | | | | |
| GO:0003823 | antigen binding | molecular function | | 3.4493E-05 | | | 0.63004 | | ENSSSCG00000010044, AZGP1 |
| GO:0033189 | response to vitamin A | biological process | | 0.00024489 | | | 1 | | RBP1 |
| GO:0002138 | retinoic acid biosynthetic process | biological process | | 0.00045071 | | | 1 | | RBP1 |
| GO:0016102 | diterpenoid biosynthetic process | biological process | | 0.00045071 | | | 1 | | RBP1 |
| GO:0016114 | terpenoid biosynthetic process | biological process | | 0.00066941 | | | 1 | | RBP1 |
| GO:0006776 | vitamin A metabolic process | biological process | | 0.00092333 | | | 1 | | RBP1 |
| GO:0002376 | immune system process | biological process | | 0.0014465 | | | 1 | | ENSSSCG00000010044, AZGP1, RBP1 |
| GO:0042571 | immunoglobulin complex circulating | cellular component | | 0.0017087 | | | 1 | | ENSSSCG00000010044 |
| GO:0042573 | retinoic acid metabolic process | biological process | | 0.0018139 | | | 1 | | RBP1 |
| GO:0030852 | regulation of granulocyte differentiation | biological process | | 0.0019276 | | | 1 | | RBP1 |
| GO:0034987 | immunoglobulin receptor binding | molecular function | | 0.0021166 | | | 1 | | ENSSSCG00000010044 |
| GO:0006958 | complement activation classical pathway | biological process | | 0.0023897 | | | 1 | | ENSSSCG00000010044 |
| GO:0019814 | immunoglobulin complex circulating | cellular component | | 0.0024088 | | | 1 | | ENSSSCG00000010044 |
| GO:0033273 | response to vitamin | biological process | | 0.002841 | | | 1 | | RBP1 |
| GO:0006910 | phagocytosis recognition | biological process | | 0.002934 | | | 1 | | ENSSSCG00000010044 |
| **GO enrichment analysis (top 15 terms) of the downregulated genes in immunocastrated compared to surgically castrated pigs** | | | | | | | | | |
| GO:0006909 | phagocytosis | biological process | | 0.00025267 | | | 0.45486 | | TGM2, PTX3 |
| GO:0030684 | preribosome | cellular component | | 0.00030055 | | | 0.45486 | | RRP1B, RRP9 |
| GO:0001872 | (1->3)-beta-D-glucan binding | molecular function | | 0.00032373 | | | 0.45486 | | PTX3 |
| GO:0044866 | modulation by host of viral exo-alpha-sialidase activity | biological process | | 0.00032373 | | | 0.45486 | | PTX3 |
| GO:0044867 | modulation by host of viral catalytic activity | biological process | | 0.00032373 | | | 0.45486 | | PTX3 |
| GO:0044868 | modulation by host of viral molecular function | biological process | | 0.00032373 | | | 0.45486 | | PTX3 |
| GO:0044869 | negative regulation by host of viral exo-alpha-sialidase activity | biological process | | 0.00032373 | | | 0.45486 | | PTX3 |
| GO:0044870 | modulation by host of viral glycoprotein metabolic process | biological process | | 0.00032373 | | | 0.45486 | | PTX3 |
| GO:0044871 | negative regulation by host of viral glycoprotein metabolic process | biological process | | 0.00032373 | | | 0.45486 | | PTX3 |
| GO:0052199 | negative regulation of catalytic activity in other organism involved in symbiotic interaction | biological process | | 0.00032373 | | | 0.45486 | | PTX3 |
| GO:0052403 | negative regulation by host of symbiont catalytic activity | biological process | | 0.00032373 | | | 0.45486 | | PTX3 |
| GO:1903015 | regulation of exo-alpha-sialidase activity | biological process | | 0.00032373 | | | 0.45486 | | PTX3 |
| GO:1903016 | negative regulation of exo-alpha-sialidase activity | biological process | | 0.00032373 | | | 0.45486 | | PTX3 |
| GO:0005730 | nucleolus | cellular component | | 0.00055521 | | | 0.62236 | | RRP12, RRP1B, PTX3 |
| GO:0046790 | virion binding | molecular function | | 0.00064737 | | | 0.62236 | | PTX3 |
|  |  |  | |  | | |  | |  |
| **GO enrichment analysis (top 15 terms) of the upregulated genes in entire males compared to surgically castrated pigs** | | | | | | | | | |
| GO:0031012 | extracellular matrix | cellular component | 2.7035E-08 | | | 0.00049383 | | SERPINF1, DCN, AEBP1, OMF, F3, ASPN, COL6A3, SPON1, POSTN | |
| GO:0044421 | extracellular region part | cellular component | 8.5021E-07 | | | 0.007765 | | C7, HSPH1, ENSSSCG00000021379, DCN, S100A6, C1QTNF6, PAPPA2, AEBP1, COL6A3, SERPINF1, HSPA8, PPL, OMD, F3, ASPN, POSTN | |
| GO:0005615 | extracellular space | cellular component | 1.4542E-06 | | | 0.0088544 | | POSTN, COL6A3, ENSSSCG00000030325, OMD, F3, AEBP1, DCN, ENSSSCG00000021379, HSPA8, SERPINF1 | |
| GO:0005201 | extracellular matrix structural constituent | molecular function | 2.6932E-06 | | | 0.0098635 | | ENSSSCG00000021943, ENSSSCG00000022000, ENSSSCG00000017581, COL1A2 | |
| GO:0005576 | extracellular region | cellular component | 0.0000027 | | | 0.0098635 | | COL6A3, C1QTNF6, PAPPA2, AEBP1, S100A6, DCN, ENSSSCG00000021379, C7, HSPH1, POSTN, ASPN, OMD, F3, PPL, HSPA8, SERPINF1 | |
| GO:0070206 | protein trimerization | biological_process | 3.828E-06 | | | 0.011654 | | ENSSSCG00000021379, SCARA5, C1QTNF6 | |
| GO:0070208 | protein heterotrimerization | biological_process | 1.6767E-05 | | | 0.043753 | | ENSSSCG00000021379, C1QTNF6 | |
| GO:0005518 | collagen binding | molecular_function | 7.4242E-05 | | | 0.16951 | | ENSSSCG00000021379, ASPN, DCN | |
| GO:0005581 | collagen trimer | cellular component | 8.9689E-05 | | | 0.18203 | | COL6A3, ENSSSCG00000017581, C1QTNF6 | |
| GO:1900744 | regulation of p38MAPK cascade | biological_process | 0.0003919 | | | 0.71112 | | HSPH1, ENSSSCG00000009585 | |
| GO:0038066 | p38MAPK cascade | biological_process | 0.0005086 | | | 0.71112 | | ENSSSCG00000009585, HSPH1 | |
| GO:0070062 | extracellular exosome | cellular component | 0.00053668 | | | 0.71112 | | S100A6, AEBP1, F3, PAPPA2, OMD, COL6A3, C7, HSPH1, SERPINF1, PPL | |
| GO:0065010 | extracellular membrane-bounded organelle | cellular component | 0.00053837 | | | 0.71112 | | COL6A3, S100A6, F3, OMD, PAPPA2, AEBP1, PPL, SERPINF1, C7, HSPH1 | |
| GO:1903561 | extracellular vesicle | cellular component | 0.00058216 | | | 0.71112 | | PPL, C7, HSPH1, SERPINF1, COL6A3, AEBP1, F3, PAPPA2, OMD, S100A6 | |
| GO:0043230 | extracellular organelle | cellular component | 0.00058397 | | | 0.71112 | | HSPH1, C7, SERPINF1, PPL, S100A6, AEBP1, PAPPA2, F3, OMD, COL6A3 | |
| **GO enrichment analysis (top 15 terms) of the downregulated genes in entire males compared to surgically castrated pigs** | | | | | | | | | |
| GO:0014734 | skeletal muscle hypertrophy | biological process | 0.00043595 | | | 0.53033 | | ENSSSCG00000015481 | |
| GO:0038129 | ERBB3 signaling pathway | biological process | 0.00043595 | | | 0.53033 | | ENSSSCG00000015481 | |
| GO:0038133 | ERBB2-ERBB3 signaling pathway | biological_process | 0.00043595 | | | 0.53033 | | ENSSSCG00000015481 | |
| GO:0001872 | (1->3)-beta-D-glucan binding | molecular_function | 0.00043595 | | | 0.53033 | | PTX3 | |
| GO:0044866 | modulation by host of viral exo-alpha-sialidase activity | biological_process | 0.00043595 | | | 0.53033 | | PTX3 | |
| GO:0044867 | modulation by host of viral catalytic activity | biological_process | 0.00043595 | | | 0.53033 | | PTX3 | |
| GO:0044868 | modulation by host of viral molecular function | biological_process | 0.00043595 | | | 0.53033 | | PTX3 | |
| GO:0044869 | negative regulation by host of viral exo-alpha-sialidase activity | biological_process | 0.00043595 | | | 0.53033 | | PTX3 | |
| GO:0044870 | modulation by host of viral glycoprotein metabolic process | biological_process | 0.00043595 | | | 0.53033 | | PTX3 | |
| GO:0044871 | negative regulation by host of viral glycoprotein metabolic process | biological_process | 0.00043595 | | | 0.53033 | | PTX3 | |
| GO:0052199 | negative regulation of catalytic activity in other organism involved in symbiotic interaction | biological_process | 0.00043595 | | | 0.53033 | | PTX3 | |
| GO:0052403 | negative regulation by host of symbiont catalytic activity | biological_process | 0.00043595 | | | 0.53033 | | PTX3 | |
| GO:1903015 | regulation of exo-alpha-sialidase activity | biological_process | 0.00043595 | | | 0.53033 | | PTX3 | |
| GO:1903016 | negative regulation of exo-alpha-sialidase activity | biological_process | 0.00043595 | | | 0.53033 | | PTX3 | |
| GO:0046327 | glycerol biosynthetic process from pyruvate | biological_process | 0.00043595 | | | 0.53033 | | PCK1 | |
